# PfMAP-2 is essential for male gametogenesis in the malaria parasite *Plasmodium falciparum*

**DOI:** 10.1038/s41598-020-68717-5

**Published:** 2020-07-17

**Authors:** Eva Hitz, Aurélia C. Balestra, Mathieu Brochet, Till S. Voss

**Affiliations:** 1 Department of Medical Parasitology and Infection Biology, Swiss Tropical and Public Health Institute, 4051 Basel, Switzerland; 2 University of Basel, 4001 Basel, Switzerland; 3 Department of Microbiology and Molecular Medicine, Faculty of Medicine, University of Geneva, 1211 Geneva 4, Switzerland

**Keywords:** Parasite biology, Parasite development, Parasite genetics, Functional genomics

## Abstract

In malaria parasites, male gametogenesis is a proliferative stage essential for parasite transmission to the mosquito vector. It is a rapid process involving three rounds of genome replication alternating with closed endomitoses, and assembly of axonemes to produce eight flagellated motile microgametes. Studies in *Plasmodium berghei* have highlighted tight regulation of gametogenesis by a network of kinases. The *P. berghei* MAPK homologue PbMAP-2 is dispensable for asexual development but important at the induction of axoneme motility. However, in *P. falciparum*, causing the most severe form of human malaria, PfMAP-2 was suggested to be essential for asexual proliferation indicating distinct functions for MAP-2 in these two *Plasmodium* species. We here show that PfMAP-2 is dispensable for asexual growth but important for male gametogenesis in vitro*.* Similar to PbMAP-2, PfMAP-2 is required for initiating axonemal beating but not for prior DNA replication or axoneme formation. In addition, single and double null mutants of PfMAP-2 and the second *P. falciparum* MAPK homologue PfMAP-1 show no defect in asexual proliferation, sexual commitment or gametocytogenesis. Our results suggest that MAPK activity plays no major role in the biology of both asexual and sexual blood stage parasites up until the point of male gametogenesis.

## Introduction

In the human host, *Plasmodium* spp. parasites undergo repeated rounds of asexual replication within human red blood cells (RBCs) thereby causing malaria symptoms. A small subset of parasites, however, undergo sexual commitment and differentiate into gametocytes, which are the human-to-mosquito transmissible parasite forms. Previous studies on *P. falciparum* sexual commitment and development have shown that a single intra-erythrocytic schizont gives rise to either only asexual or only sexual progeny^1,2^. However, recent research demonstrated that schizonts can also generate mixed asexual and sexual progeny thus promoting the idea of same cycle sexual conversion in ring stage parasites^3^. Following commitment to sexual development, gametocytes mature over 10 to 12 days and five distinct morphological stages into mature stage V gametocytes. Male-specific marker genes can be detected as early as in stage I/II gametocytes^4^, whereas morphological differentiation of male and female gametocytes using Giemsa-stained blood smears is only evident in later stages IV and V. In vivo, stage I–IV gametocytes sequester in tissues including the bone marrow and are thus absent from blood circulation^5–9^. In contrast, mature stage V gametocytes re-enter the bloodstream from where they can eventually be taken up by a female *Anopheles* mosquito during a blood meal^10^. Upon ingestion by an *Anopheles* vector, gametocytes encounter major environmental changes in the mosquito midgut. A drop in temperature, a rise in pH and the presence of the mosquito factor xanthurenic acid (XA) trigger the egress of gametocytes from the infected RBC (iRBC) and gamete development^11–15^. Whereas one female gametocyte develops into a single macrogamete, one male gametocyte gives rise to eight flagellated motile microgametes. Gametogenesis is linked to intracellular mobilisation of Ca^2+^, which in male gametocytes activates three rounds of rapid replication of DNA followed by endomitosis^16^. During this process, parasites also egress from the RBC and start axoneme biosynthesis. In the last phase of male gametogenesis, axoneme mobility is initiated and male gametes exit into the environment in a process termed exflagellation. At this point, the motile male gametes are still attached to the residual body and bind neighbouring erythrocytes, thus generating so-called exflagellation centres that are visible by bright-field microscopy^17–19^. Subsequently, in the mosquito midgut, one female macrogamete fuses with one male microgamete to generate a zygote. Further development results in a motile ookinete that traverses the mosquito midgut epithelium to form a sessile oocyst. The oocyst undergoes sporogony resulting in the generation of thousands of sporozoites that, upon release from the oocyst, infect the mosquito salivary glands from where they are injected into a new host during a next blood meal.

Studies mainly performed in *P. berghei*, a malaria parasite infecting rodents, have identified several kinases as important components in different steps of male gametogenesis. For instance, the cGMP-dependent protein kinase G (PKG) was shown to be essential for Ca^2+^ mobilisation upon gametocyte activation by environmental triggers^20,21^. Following Ca^2+^ mobilisation, the action of the calcium-dependent protein kinase 4 (CDPK4) is essential for microgamete development at three different steps including the initiation of axoneme motility^18,22,23^. In addition, other calcium-dependent protein kinases (CDPK1 and CDPK2) play important roles at different steps of male gametogenesis^24–26^.

In 2005, three different studies identified the atypical *P. berghei* mitogen-activated protein kinase (MAPK) PbMAP-2 as an important component in male gametogenesis^27–29^. MAP-2 together with MAP-1 represent the only two homologues of eukaryotic MAPKs identified in *Plasmodium* spp.^30–33^. In various eukaryotes ranging from yeast to humans, the MAPK signalling pathway was shown to be involved in essential cellular processes including cell differentiation, proliferation as well as survival^34,35^. In *P. berghei*, PbMAP-2 is dispensable for asexual proliferation of blood stage parasites. However, in PbMAP-2 knockout (KO) parasites, a dramatic decrease in the number of exflagellating male gametocytes was observed when compared to wild type (WT) parasites^27–29^. Tewari and colleagues further scrutinized the PbMAP-2-dependent exflagellation defect and identified an important role for PbMAP-2 late in gametogenesis at the stage of DNA condensation, initiation of axoneme motility and cytokinesis^29^. As expected, due to these defects PbMAP-2 KO parasites were essentially unable to infect *Anopheles* mosquitoes^28,29^.

In contrast to *P. berghei*, the role of PfMAP-2 in *P. falciparum* parasites remains elusive as the *pfmap-2* gene was found resistant to KO attempts. It was therefore speculated that PfMAP-2 is essential for *P. falciparum* asexual proliferation and thus may have roles distinct from its function in *P. berghei* parasites^36^. Dorin-Semblat and colleagues could further show that the second *P. falciparum* MAPK, PfMAP-1, is neither essential for asexual development and gametocytogenesis in vitro nor for gametogenesis and sporogony in the mosquito vector^36^. However, the authors observed upregulation of PfMAP-2 protein expression in PfMAP-1 KO parasites and therefore suggested a mechanism through which increased PfMAP-2 kinase levels may compensate for the loss of PfMAP-1 function^36^. Such functional compensation would imply that PfMAP-1 still has an important function in asexual *P. falciparum* development in vitro. However, in *P. berghei* both MAPKs are dispensable for asexual growth and PbMAP-1/PbMAP-2 double KO parasites show no obvious phenotype in blood stage proliferation^37^.

Whereas recent research has shed light into signalling pathways and kinases involved in *P. berghei* male gamete production, knowledge on the molecular players in *P. falciparum* gametogenesis is still scarce. Here, we used CRISPR/Cas9-based gene editing to re-assess MAPK function in *P. falciparum*. We demonstrate that similar to the situation in *P. berghei*, PfMAP-1 and PfMAP-2 are dispensable for asexual proliferation, sexual commitment and gametocyte development and that PfMAP-2 shows male-specific expression and is essential for male gametogenesis.

## Results

### MAPKs are not essential for *P. falciparum* asexual development

Using reverse genetics, our first aim was to identify the function of PfMAP-2 in *P. falciparum* asexual and sexual development. Therefore, using a single plasmid CRISPR/Cas9 system^38^, we aimed at generating a PfMAP-2 KO parasite line, although previous research reported unsuccessful KO attempts^36^. Using this approach, correct editing of the locus would result in the replacement of the *pfmap-2* gene with a blasticidin-S-deaminase (BSD)-expressing resistance cassette. In multiple independent transfections of strain NF54, we obtained drug-resistant parasites 10–14 days after transfection. PCR on genomic DNA (gDNA) confirmed the disruption of the *pfmap-2* gene and the correct integration of the BSD resistance cassette and thus the successful generation of a NF54/MAP-2 KO line (Supplementary Fig. 1). Hence, contrary to previous research, we conclude that PfMAP-2 is not essential for asexual proliferation.

**Figure 1:**
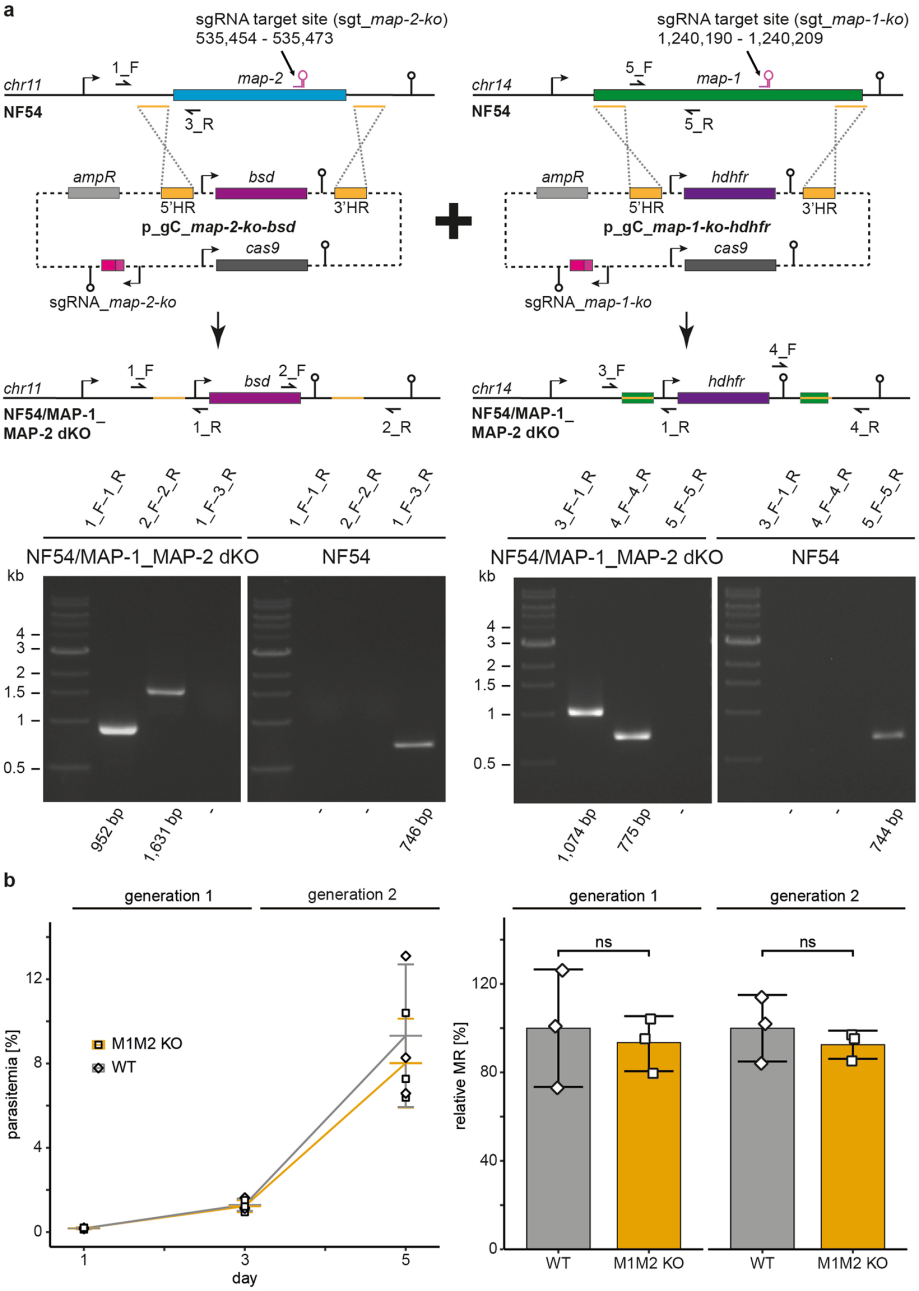
Engineering and growth assay of the MAPK double KO parasite line. (**a**) Top: Scheme depicting the CRISPR/Cas9-based gene editing approach used to generate the NF54/MAP-1_MAP-2 dKO parasite line. Schematic maps of the *pfmap-2* locus and the CRISPR/Cas9 p_gC_*map-2-ko-bsd* plasmid used to generate the NF54/MAP-2 KO line (left), that was re-transfected using the p_gC_*map-1-ko-hdhfr* plasmid to generate the NF54/MAP-1_MAP-2 dKO parasite line (right). Bottom: PCRs performed on gDNA of NF54/MAP-1_MAP-2 dKO parasites show correct editing of both *mapk* loci. PCRs performed on NF54 WT gDNA serve as controls. (**b**) Left: Flow cytometry data showing the increase in parasitemia of NF54 WT and NF54/MAP-1_MAP-2 dKO parasites over two generations. Right: Multiplication rates quantified from flow cytometry data in NF54 WT and the NF54/MAP-1_MAP-2 dKO parasites in both generations. Values show the means ± SD of three biological replicates with individual data points represented by open squares. ns, not significant (unpaired two-tailed Student’s t test); MR, multiplication rate; WT, NF54 WT; M1M2 KO, NF54/MAP-1_MAP-2 dKO.

In addition, using the same approach, we also generated a PfMAP-1 KO parasite line (NF54/MAP-1 KO) serving as a control for further experiments. NF54/MAP-1 KO parasites could be readily obtained as previously described^36^ (Supplementary Fig. 1). To test if both PfMAPKs are dispensable for asexual growth, we tried to generate a PfMAP-1/PfMAP-2 double KO (dKO) parasite line. We therefore re-transfected the NF54/MAP-2 KO line using a plasmid targeting the *pfmap-1* locus for disruption, this time replacing the *pfmap-1* coding sequence with an expression cassette encoding the drug resistance marker human dihydrofolate reductase (hDHFR) (Fig. 1a). NF54/MAP-1_MAP-2 dKO parasites could be readily obtained and disruption of both *mapk* loci was confirmed by PCR on gDNA (Fig. 1a). To identify whether the absence of both *P. falciparum* MAPKs causes a defect in parasite growth, we performed parasite multiplication assays comparing NF54 WT and NF54/MAP-1_MAP-2 dKO parasites. As shown in Fig. 1b, parasite growth was not affected in the MAPK dKO parasites. Taken together, our data demonstrate that both *P. falciparum* MAPKs are dispensable for asexual proliferation of blood stage *P. falciparum* parasites in vitro and that PfMAP-2 is not required to compensate for the loss of PfMAP-1 function.

### MAPKs are not essential for sexual commitment and gametocytogenesis

**Figure 2:**
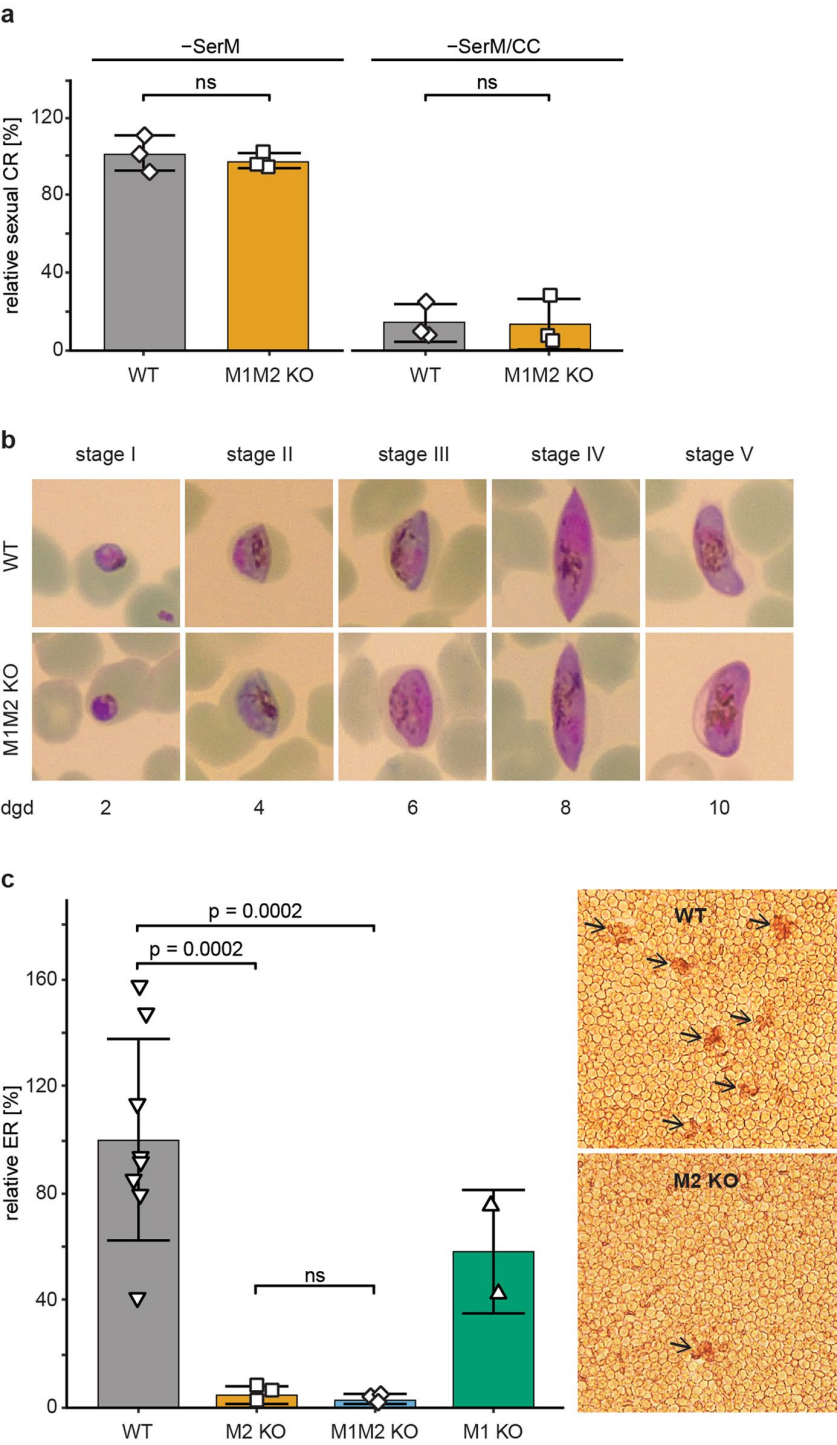
Sexual commitment, sexual development and exflagellation of MAPK double KO parasites. (**a**) Relative sexual commitment rates of NF54 WT and NF54/MAP-1_MAP-2 dKO parasites cultured in serum-depleted medium either in presence (− SerM/CC) or absence (− SerM) of choline chloride quantified using Pfs16-positivity counts from α-Pfs16 IFA (> 200 iRBCs counted per experiment). Values show the means ± SD of three biological replicates with individual data points represented by open squares. ns, not significant; (unpaired two-tailed Student’s t test). CR, commitment rate; WT, NF54 WT; M1M2 KO, NF54/MAP-1_MAP-2 dKO. (**b**) Representative images of Giemsa-stained parasites in thin blood smears showing development of NF54 WT and NF54/MAP-1_MAP-2 dKO gametocytes over 10 days and the five (I-V) distinct morphological stages. 1,000 × magnification. dgd, day of gametocyte development; WT, NF54 WT; M1M2 KO, NF54/MAP-1_MAP-2 dKO. (**c**) Left: Relative exflagellation rates of NF54/MAP-2 KO, NF54/MAP-1_MAP-2 dKO and NF54/MAP-1 KO gametocytes compared to NF54 WT control parasites. Values show the means ± SD of two (NF54/MAP-1 KO), eight (NF54 WT) or three (NF54/MAP-2 KO and NF54/MAP-1_MAP-2 dKO) biological replicates with individual data points represented by open symbols. Significant differences are indicated (p-value, unpaired two-tailed Student’s t test). ns, not significant. Right: Representative images of the number of exflagellation centres (indicated by black arrows) observed in NF54 WT and NF54/MAP-2 KO parasites with comparable gametocytemia (NF54 WT: 2.2%; NF54/MAP-2 KO: 1.8%) by live microscopy (200 × magnification). WT, NF54 WT; M1 KO, NF54/MAP-1 KO; M2 KO, NF54/MAP-2 KO; M1M2 KO, NF54/MAP-1_MAP-2 dKO; ER, exflagellation rate.

PfMAP-1 was shown to be dispensable for gametocytogenesis in vitro as well as for gametogenesis and sporogony in the mosquito vector^36^. To investigate the effect of the absence of both MAPKs on gametocyte development, we induced sexual commitment in synchronous NF54/MAP-1_MAP-2 dKO parasite cultures at 18–24 h post invasion (hpi) using serum-free medium (− SerM) as previously described^39^. Parasites cultured on − SerM supplemented with 2 mM choline chloride (a metabolite supressing sexual commitment) (− SerM/CC) served as control^39^. As a proxy for the sexual commitment rate, the percentage of parasites expressing the gametocyte-specific marker Pfs16 in the progeny was quantified by immunofluorescence assays (IFA) (Fig. 2a). Comparison of NF54 WT with the NF54/MAP-1_MAP-2 dKO parasite line did not reveal a significant difference in the sexual commitment rates under both commitment-inducing (− SerM) and control (− SerM/CC) conditions (Fig. 2a). Using Giemsa-stained thin blood smears, gametocyte development was monitored over 10 days and we could not identify any apparent morphological differences between WT and MAPK dKO gametocytes in all five stages (Fig. 2b). In summary, our results demonstrate the dispensability of both MAPKs in regulating *P. falciparum* sexual commitment and development in vitro.

### PfMAP-2 is essential for male gametogenesis

In *P. berghei* parasites, PbMAP-2 was previously reported to be essential for male gametogenesis and further sexual development in the mosquito vector^27–29^. In light of this function in *P. berghei*, we investigated whether PfMAP-2 KO gametocytes also show a defect in male gametogenesis by assessing exflagellation rates (ERs). Gametogenesis was activated in mature stage V gametocytes using a drop in temperature (from 37 °C to room temperature/22 °C) and supplementation of the culture medium with XA^40^. Exflagellation centres formed by male gametes were observed and quantified using bright-field microscopy (Fig. 2c). Using total RBC and gametocytemia counts, the ER was calculated as the number of exflagellating parasites per total number of gametocytes. Our experiments revealed that compared to WT gametocytes (100% ± 37.6), parasites devoid of the PfMAP-2 kinase displayed a dramatic decrease in the relative number of exflagellation centres formed (4.9% ± 3.3) (p = 0.0002; unpaired two-tailed Student’s t test) (Fig. 2c). Despite this striking exflagellation defect, a minority of male PfMAP-2 KO gametocytes was still able to exflagellate. Similarly, this residual exflagellation capacity was also observed in *P. berghei* PbMAP-2 KO parasites^28,29^. It was speculated that these rare exflagellation events might be attributed to the action of PbMAP-1 partially compensating the loss of PbMAP-2 function^28,29^. To investigate this hypothesis, we performed exflagellation assays on both the PfMAP-1 KO and the MAPK dKO parasite lines. As previously reported, PfMAP-1 KO parasites showed no major exflagellation defect when compared to WT parasites (58% ± 23) (mean of two biological replicate experiments) (Fig. 2c). Parasites lacking both *P. falciparum* MAPKs had a similarly pronounced exflagellation defect as the PfMAP-2 KO line and again a small proportion of male gametocytes was still able to exflagellate (3% ± 1.6 compared to WT parasites) (p = 0.0002; unpaired two-tailed Student’s t test). Hence, the residual exflagellation observed in male PfMAP-2 KO gametocytes cannot be explained by partial functional compensation through PfMAP-1. Together, these results demonstrate that PfMAP-2, but not PfMAP-1, has an important role in male gametogenesis in vitro, in accord with previous findings in *P. berghei* PbMAP-2 KO parasites^27–29^.

### PfMAP-2 is specifically expressed in male gametocytes

**Figure 3:**
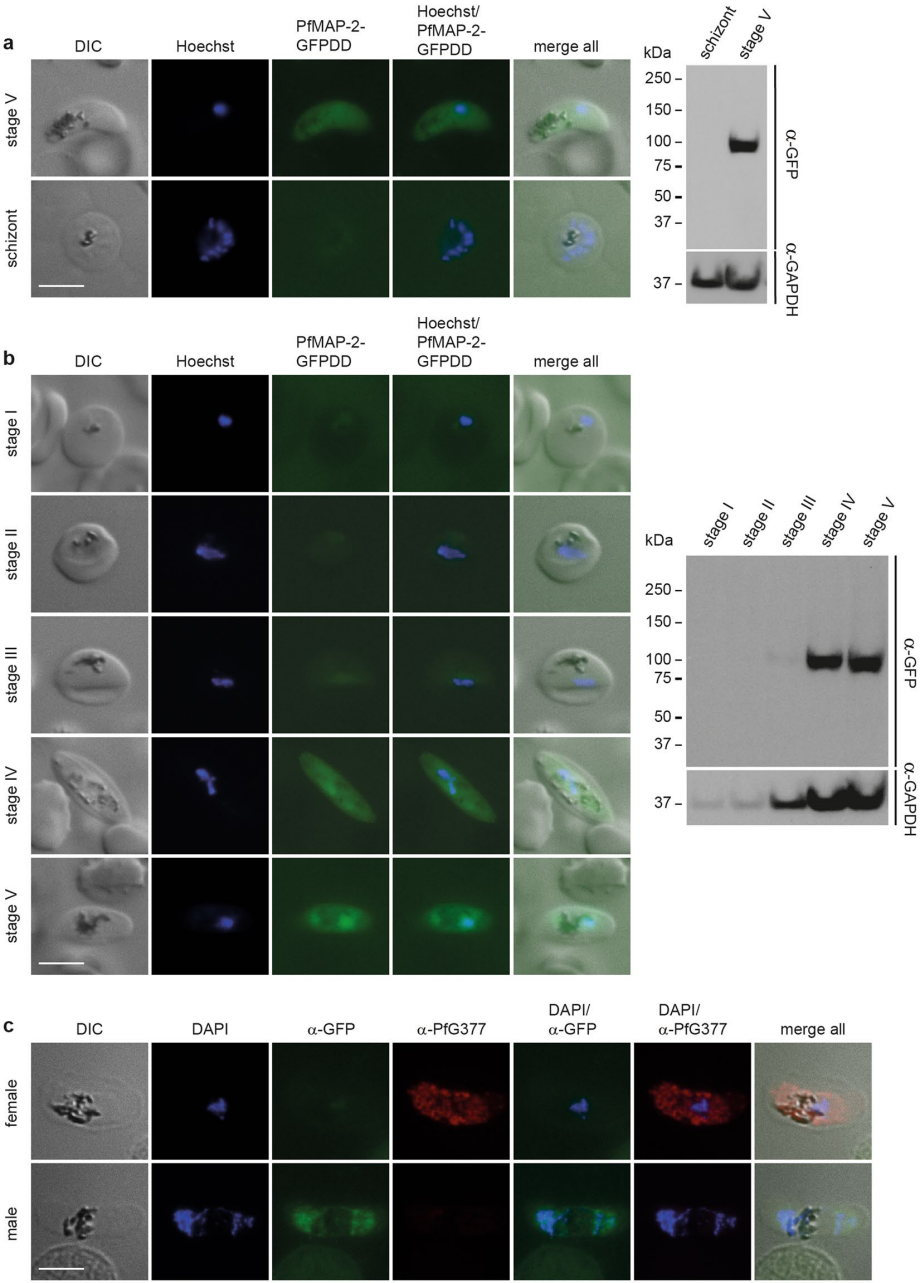
Expression and localisation of PfMAP-2 in NF54/MAP-2GFPDD parasites. (**a**) Expression and localisation of PfMAP-2 in late schizonts (40–48 hpi) and stage V gametocytes assessed by live cell fluorescence imaging and Western blot. Representative live cell fluorescence images are shown. Scale bar = 5 µm. For the Western blot, parasitemia/gametocytemia was determined by inspection of Giemsa-stained blood smears to ensure loading of equal parasite numbers per lane. MW PfMAP-2GFPDD = 97.9 kDa. MW PfGAPDH = 36.6 kDa (control). (**b**) Expression and localisation of PfMAP-2 in stage I to stage V gametocytes by live cell fluorescence imaging and Western blot. Representative live cell fluorescence images are shown. Scale bar = 5 µm. For the Western blot, gametocytemia was determined by inspection of Giemsa-stained blood smears to ensure loading of equal parasite numbers per lane. MW PfMAP-2GFPDD = 97.9 kDa. MW PfGAPDH = 36.6 kDa (control). (**c**) Representative images of the expression of PfMAP-2GFPDD and PfG377 in stage V male and female gametocytes assessed by IFA. Scale bar = 5 µm. See also Supplementary Fig. 2.

Transcriptomic and proteomic data suggest that the MAP-2 kinase is specifically expressed in male gametocytes both in *P. berghei* and *P. falciparum* parasites^27,41–43^. However, in vivo expression of the protein in sexual stages has neither been assessed in *P. berghei* nor in *P. falciparum* and other studies proposed expression of PfMAP-2 also in asexual blood stage parasites^36,44^. To monitor PfMAP-2 kinase expression, we used a two-plasmid CRISPR/Cas9 gene editing approach^38^ to generate a parasite line expressing PfMAP-2 C-terminally tagged with the fluorescent marker GFP fused to a FKBP destabilization domain (DD) that facilitates modulating protein expression levels using the small molecule ligand Shield-1^45,46^ (NF54/MAP-2GFPDD) (Supplementary Fig. 2). PCR on gDNA of the NF54/MAP-2GFPDD conditional knockdown (cKD) parasite line confirmed correct editing of the locus (Supplementary Fig. 2). This PfMAP-2 cKD parasite line allows for protein visualization under DD-stabilizing conditions (+ Shield-1) by fluorescence microscopy and further provides the opportunity to investigate the effect of conditional PfMAP-2 depletion (− Shield-1) on parasite biology in comparison to the isogenic control population (+ Shield-1). To determine the stage-specific expression of PfMAP-2GFPDD, we performed live cell fluorescence imaging and Western blot analysis on both asexual parasites and gametocytes under protein-stabilizing (+ Shield-1) conditions. Western blot analysis revealed expression of PfMAP-2 in stage V gametocytes. In contrast, we did not observe PfMAP-2 expression in trophozoite and schizont stage parasites (Fig. 3a and Supplementary Figs. 2 and 5). Live cell fluorescence imaging of PfMAP-2GFPDD confirmed these findings (Fig. 3a and Supplementary Fig. 2).

To further specify the expression of PfMAP-2 during sexual stage development, we performed both Western blot analysis and live cell fluorescence imaging at all five stages of gametocytogenesis. Both techniques consistently showed peak expression of PfMAP-2 in stage IV and V gametocytes (Fig. 3b and Supplementary Fig. 5). Whereas a weak PfMAP-2GFPDD signal was detected in stage III gametocytes, the PfMAP-2 protein was not identified in gametocyte stages I and II (Fig. 3b and Supplementary Fig. 5). With regard to subcellular localization, the PfMAP-2 protein was detected in the parasite cytoplasm and nucleus in both stage IV and V gametocytes (Fig. 3b).

Interestingly, our live cell fluorescence imaging experiments detected expression of PfMAP-2 only in a subset (approx. 50%) of stage IV and V gametocytes. Since previous studies indicated that PfMAP-2 is specifically expressed in male gametocytes^27,41–43^, we investigated whether the PfMAP-2GFPDD-positive parasites indeed represented male gametocytes. To this end, we performed IFAs using antibodies against GFP and the female gametocyte-specific protein PfG377^47^. Notably, all gametocytes positively labelled for PfMAP-2GFPDD expression were identified as PfG377-negative and similarly PfG377-positive gametocytes were found to be GFP-negative (Fig. 3c and Supplementary Fig. 2). Quantification of the GFP- and the PfG377-positive cells in biological triplicates revealed a 1:1 ratio of male/female gametocytes (male: 49.7% ± 4.2; female: 50.3% ± 4.2).

Together, our data demonstrate that the PfMAP-2 kinase is predominantly expressed in late stage (IV and V) gametocytes but not, or only at low levels, in asexual stages and early stage gametocytes. In addition, we confirm at the single cell level that PfMAP-2 is indeed expressed in a male gametocyte-specific manner.

### PfMAP-2 is not essential for male gametocytogenesis

**Figure 4:**
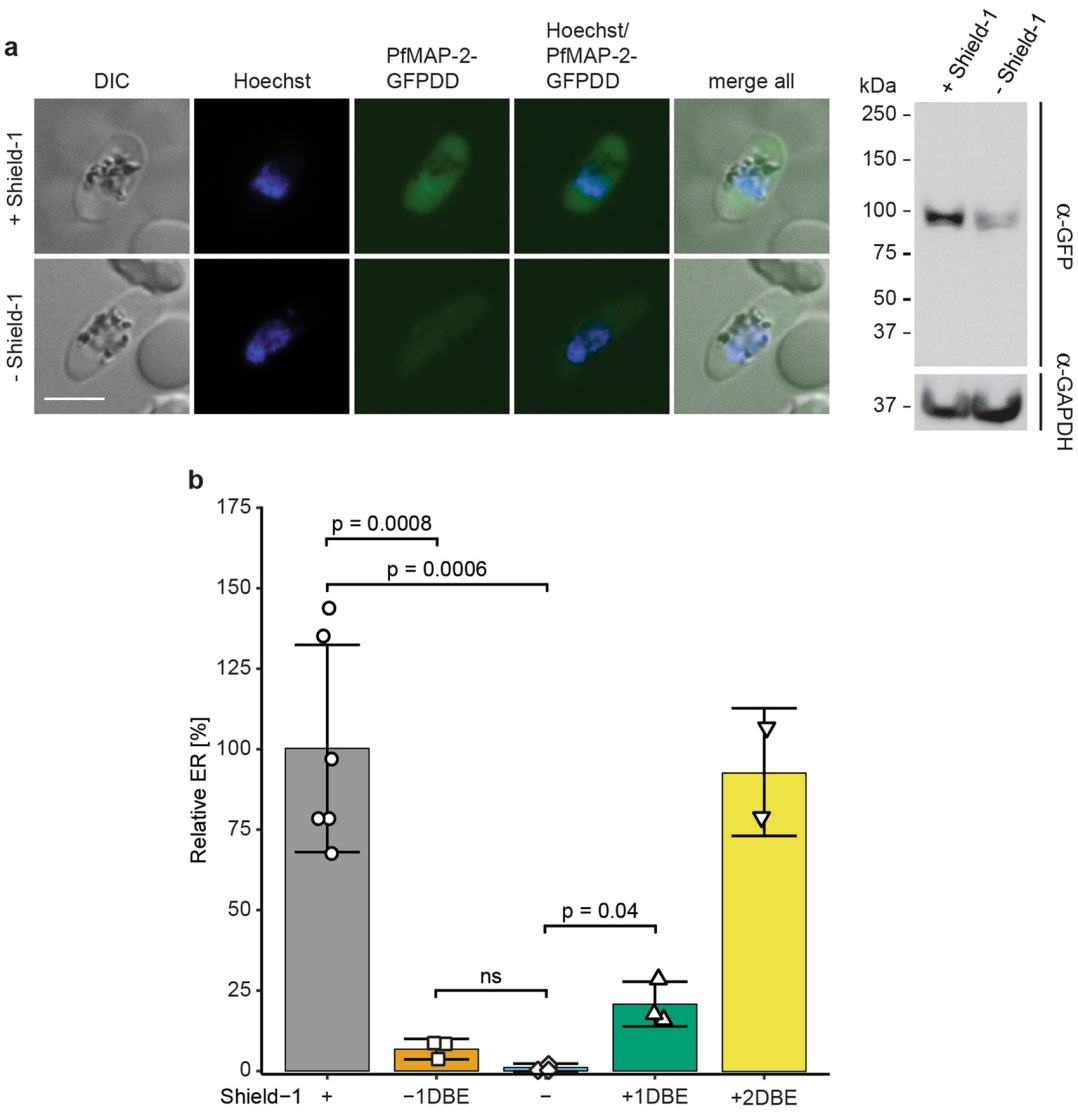
PfMAP-2 depletion in NF54/MAP-2GFPDD parasites prevents exflagellation. (**a**) Expression of PfMAP-2 in stage V gametocytes cultured in presence or absence of Shield-1 by live cell fluorescence imaging and Western blot. Parasites were split (± Shield-1) 24 h prior to the analysis. Representative live cell fluorescence images are shown. Scale bar = 5 µm. For the Western blot, gametocytemia was determined by inspection of Giemsa-stained blood smears to ensure loading of equal parasite numbers per lane. MW PfMAP-2GFPDD = 97.9 kDa. MW PfGAPDH = 36.6 kDa (control). (**b**) Relative exflagellation rates of NF54/MAP-2GFPDD gametocytes cultured constantly in presence ( +) or absence (−) of Shield-1, or where Shield-1 has been removed one day (24 h) before assessing exflagellation rates (-1DBE) or replenished two days (48 h) and one day (24 h) before assessing exflagellation rates (+ 2DBE and + 1DBE, respectively). Values show the means ± SD of two (+ 2DBE), three (−; − 1DBE; + 1DBE) or six ( +) biological replicates with individual data points represented by open symbols. Significant differences are indicated (p-value, unpaired two-tailed Student’s t test). ns, not significant. ER, exflagellation rate.

Next, we tested the functionality and efficiency of the conditional expression system by comparing NF54/MAP-2GFPDD parasites cultured under protein-stabilizing (+ Shield-1) and protein-degrading (− Shield-1) conditions. For this purpose, stage V gametocytes constantly cultured on Shield-1 were split and Shield-1 was removed from one of the paired cultures. 24 h later, we detected the expression of the PfMAP-2GFPDD protein by live cell fluorescence imaging and Western blot analysis. Efficient degradation of PfMAP-2GFPDD was observed in stage V gametocytes cultured in absence of Shield-1 (− Shield-1) compared to the control population (+ Shield-1) using both techniques (Fig. 4a and Supplementary Fig. 6). To confirm the essential role for PfMAP-2 in male gametogenesis, we performed exflagellation assays comparing stage V gametocytes constantly cultured on Shield-1 (+ Shield-1) with the paired population from which Shield-1 was removed 24 h earlier (− Shield-1) (i.e. one day before exflagellation; − 1DBE). We identified a dramatic exflagellation defect in − 1DBE gametocytes (6.8% ± 3.2), compared to gametocytes cultured in presence of Shield-1 (100% ± 33.9) (p = 0.0008; unpaired two-tailed Student’s t test) (Fig. 4b). The low exflagellation rate found in − 1DBE gametocytes is comparable to that observation for PfMAP-2 KO parasites (4.9% ± 3.3) (Fig. 2c). Since removal of Shield-1 for only 24 h causes a dramatic exflagellation defect, we assume that this phenotype is linked to a direct requirement for PfMAP-2 during gametogenesis rather than a prior cryptic role for PfMAP-2 during gametocytogenesis that cannot be detected by microscopic observations (Fig. 2b). To further confirm this hypothesis, we triggered PfMAP-2GFPDD depletion in asexual ring stage parasites before induction of sexual commitment and constantly cultured the resulting gametocytes in absence of Shield-1 (− Shield-1) until mature stage V gametocytes were observed. One day (24 h) before performing exflagellation assays, we split these PfMAP-2GFPDD-depleted stage V gametocyte cultures and rescued PfMAP-2GFPDD expression in one of the paired populations by addition of Shield-1 (+ Shield-1 1DBE). Indeed, stage V gametocytes allowed to re-express PfMAP-2GFPDD for 24 h (+ Shield-1 1DBE) showed a significantly increased exflagellation rate (20.8% ± 6.9) compared to gametocytes cultured constantly in absence of Shield-1 (− Shield-1) (1% ± 1.2) (p = 0.04; unpaired two-tailed Student’s t test) (Fig. 4b). However, the + Shield-1 1DBE gametocytes only reached about 20% of the exflagellation rate observed for gametocytes constantly cultured in presence of Shield-1 (+ Shield-1) (100% ± 32.1) (Fig. 4b). Interestingly, when PfMAP-2GFPDD-depleted stage V gametocytes were allowed to re-express PfMAP-2 for 48 h prior to performing exflagellation assays (+ Shield-1 2DBE), exflagellation rates reached values similar to those determined in control gametocytes (+ Shield-1) (92.6% ± 20) (Fig. 4b).

Hence, by analysing the PfMAP-2GFPDD cKD mutant we could confirm the essential role of PfMAP-2 in male gametogenesis, since conditional depletion of PfMAP-2 yielded an exflagellation defect comparable to the one observed in PfMAP-2 KO parasites. In addition, our results suggest that the requirement for PfMAP-2 function for successful exflagellation of male gametocytes is based on the action of this kinase during gametogenesis rather than any prior preparatory processes during male gametocytogenesis.

### PfMAP-2 is required to initiate axoneme beating

The defect in exflagellation observed in the absence of PfMAP-2 confirmed the essential function of PbMAP-2 during male gametogenesis in *P. berghei*. We therefore carried out a more detailed phenotypic analysis of the PfMAP-2GFPDD cKD line by removing Shield-1 in stage V gametocytes 24 h prior to phenotyping assays.

Mature *P. falciparum* gametocytes are crescent-shaped but rapidly become spherical upon stimulation by XA, a process known as “rounding up”^48^. Microscopic observation and quantification of activated gametocytes demonstrated that PfMAP-2GFPDD gametocytes cultured in presence (+ Shield-1, 99.7% ± 0.6) and absence (− Shield-1, 98% ± 1) of Shield-1 showed no difference in rounding up when activated with XA-containing medium (values represent the means ± SD of three biological replicates, 100 cells were counted per experiment, paired two-tailed Student’s t test).

**Figure 5:**
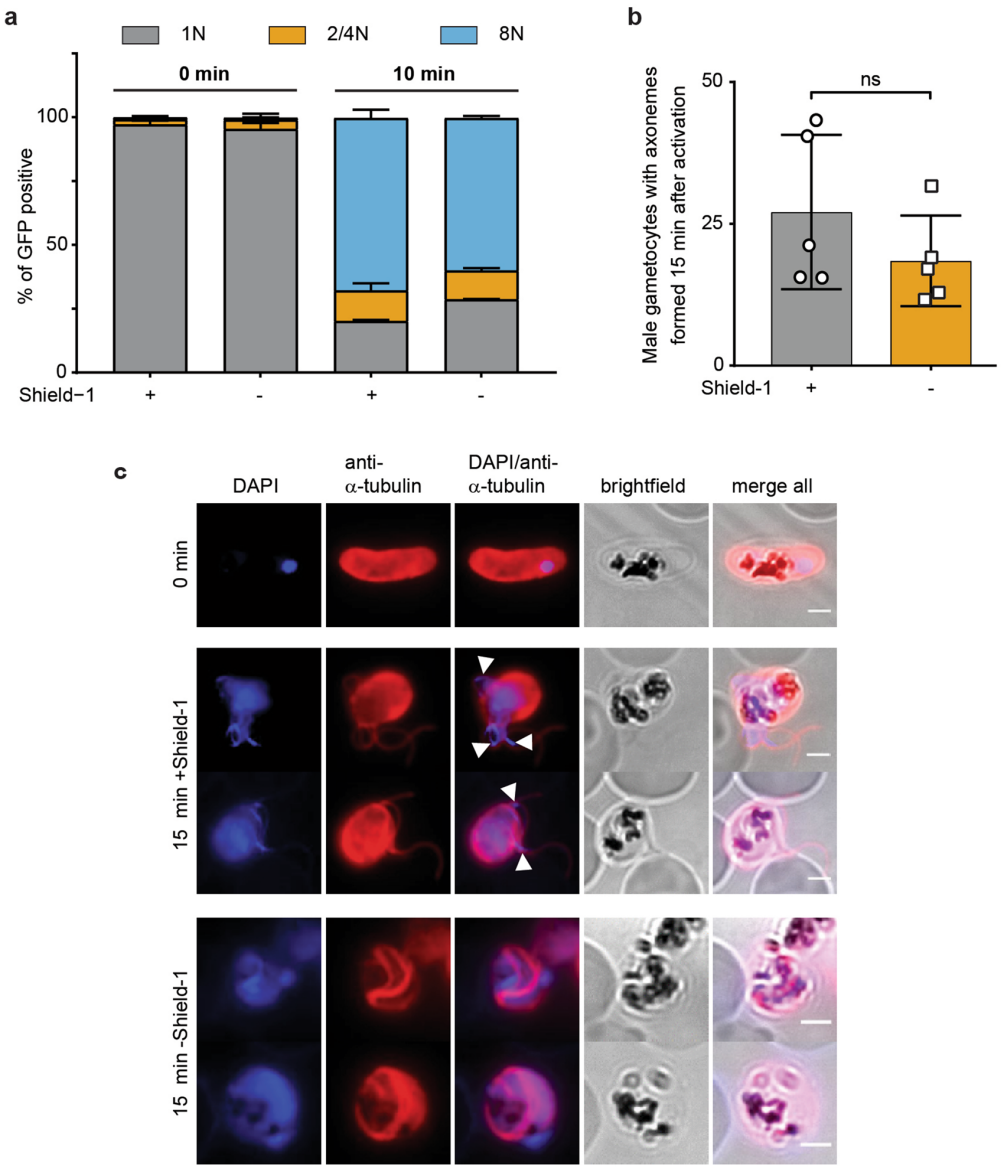
Detailed phenotyping of the exflagellation defect in NF54/MAP-2GFPDD parasites. (**a**) Ploidy of GFP-positive microgametocytes 0 and 10 min after activation of gametogenesis under protein-stabilizing (+ Shield-1) and -degrading (− Shield-1) conditions assessed by flow cytometry. Parasites were split (± Shield-1) 24 h before analysis. Values show the means ± SD of three biological replicates. (**b**) Number of male gametocytes that form axonemes 15 min after activation of gametogenesis, determined from anti-α-tubulin IFAs. Protein-stabilizing (+ Shield-1) and -degrading (− Shield-1) culturing conditions are compared. Parasites were split (± Shield-1) 24 h before analysis. Values show the means ± SD of five biological replicates with individual data points represented by open symbols. ns, not significant (paired two-tailed Student’s t test). (**c**) Representative images of anti-α-tubulin IFAs showing axoneme formation 15 min after activation of gametogenesis. Protein-stabilizing (+ Shield-1) and -degrading (− Shield-1) culturing conditions are compared. White arrowheads indicate incorporation of DNA into newly forming microgametes exclusively in parasites cultured under protein-stabilizing (+ Shield-1) conditions. Parasites were split (± Shield-1) 24 h before probing. Scale bar = 2 µm.

We then asked whether PfMAP-2 is required to replicate the genome of microgametocytes three times. To do so, cells were stained with the Vybrant DyeCycle Violet DNA dye and fluorescence intensity of non-activated gametocytes and gametocytes activated for 10 min was determined by flow cytometry. Note that due to residual GFP-fluorescence detectable by flow cytometry in gametocytes cultured in absence of Shield-1, those microgametocytes depleted for PfMAP-2GFPDD expression could still be quantified based on fluorescence by gating of GFP-positive cells (Supplementary Fig. 3). No difference in DNA content was detected between microgametocytes cultured in presence and absence of Shield-1, indicating that PfMAP-2 is not required for genome replication in male gametocytes (Fig. 5a and Supplementary Fig. 3). Immunofluorescence labelling with an α-tubulin antibody indicated that axoneme formation is also not affected in PfMAP-2GFPDD-depleted male gametocytes 15 min post-activation (Fig. 5b,c). However, in PfMAP-2GFPDD-depleted cells the axonemes remained bundled around the nucleus, nuclear DNA is not incorporated into the forming microgametes and no exflagellation is observed (Fig. 5c and Supplementary Fig. 3). In contrast, male gametocytes cultured in the presence of Shield-1 began to release motile microgametes into which DNA was incorporated (Fig. 5c and Supplementary Fig. 3).

### PfMAP-2 depletion does not affect gene transcription in stage V gametocyte bulk populations

Our results demonstrate the dispensability of PfMAP-2 in male gametocytogenesis but its strict requirement for male gametogenesis. MAPKs were previously shown to indirectly or directly regulate gene expression in other eukaryotes^34,49^. Using a comparative transcriptome analysis, we tested whether PfMAP-2 depletion has an effect on gene expression in mature stage V gametocytes prior to or 10 min after activation using XA. Using the same approach, we also asked if gene expression differs between non-activated gametocytes and gametocytes 10 min post-XA treatment. Note that a previous study reported up to two-fold differential expression of several genes between non-activated gametocytes and gametes 30 min after activation by XA using quantitative real time PCR^50^. To our knowledge, however, potential transcriptional changes occurring during the first 10 min of the exflagellation process have not yet been assessed. To this end, we split NF54/MAP-2GFPDD stage V gametocytes and removed Shield-1 from one of the two paired populations. 24 h later each of the paired + Shield-1/− Shield-1 populations (stage V ON and stage V OFF) was split again and one half each was triggered for gamete activation by incubation in XA-containing medium for 10 min (stage V act. ON and stage V act. OFF) (Supplementary Fig. 4). Total RNA was then harvested from these four populations in two biological replicate experiments and relative gene expression values were quantified by two-colour microarray analysis^51,52^ (Supplementary Dataset 1). As shown in Supplementary Fig. 4, all eight transcriptomes showed almost perfect correlation in pairwise comparisons of their relative mRNA abundances (Perason’s *r* > 0.98). Furthermore, by comparison to a published reference dataset of gametocyte-specific transcripts^53^ we show that the transcriptomes determined here display a typical gametocyte-specific gene expression profile (Supplementary Fig. 4). To identify genes potentially regulated in a PfMAP-2-dependent manner, we compared the mean relative gene expression values calculated from the four control samples (+ Shield-1; stage V ON and stage V act. ON; two biological replicates each) with those calculated from the four PfMAP-2-depleted samples (− Shield-1; stage V OFF and stage V act. OFF; two biological replicates each). This analysis failed to reveal any genes displaying significant differential expression (mean fold change > 2; p < 0.01) in PfMAP-2-depleted gametocytes (− Shield-1) versus control gametocytes (+ Shield-1) (Supplementary Fig. 4). Similarly, after stratifying the data according to non-activated (pre-XA treatment) and activated (post-XA treatment) gametocytes we didn’t identify a single gene consistently up- or down-regulated > twofold in both biological replicates when comparing PfMAP-2-depleted gametocytes (− Shield-1) to control gametocytes (+ Shield-1) in either of the two conditions (Supplementary Dataset 1). We therefore conclude that (i) PfMAP-2 likely plays no major role in regulating gene expression during exflagellation but rather in phosphorylating crucial cell cycle regulators; and (ii) the process of exflagellation likely occurs in absence of any major changes in gene expression.

## Discussion

The complex life cycle of *P. falciparum* includes the asexual proliferation of parasites inside RBCs and the generation of gametocytes necessary for onward parasite transmission. Our study aimed at identifying the function of MAPKs in *P. falciparum* asexual blood stage parasites and sexual development. Using CRISPR/Cas9-based gene editing, we generated PfMAP-2 KO, PfMAP-1 KO and PfMAP-1_MAP-2 dKO parasite lines as well as a PfMAP-2 cKD cell line allowing for conditional PfMAP-2 depletion.

The *Plasmodium* MAPKs cluster with the extracellular signal-regulated kinases 1 and 2 (ERK1/ERK2) family of MAPKs, which integrate extracellular signals into cellular responses and thus have essential functions in cell proliferation and differentiation in model eukaryotes^35,54^. Hence, it is rather surprising that both *P. falciparum* MAPKs are dispensable for asexual parasite survival. However, in many aspects the *Plasmodium* kinome, including MAPK signalling, seems to diverge from that of other eukaryotes^54^. For instance, the kinome of *Plasmodium* lacks the STE group of eukaryotic protein kinases that includes the classical MAPK-activating kinases MAPKK or MEK^55^. Furthermore, whereas PfMAP-1 contains the classical TXY activation site found in eukaryotic MAPKs, PfMAP-2 lacks this motif and instead contains an atypical TSH activation motif essential for its function^30,56^. Interestingly, the NIMA-related kinase PfNEK-1 might represent an atypical functional homologue of MAPKK/MEK. PfNEK-1 phosphorylates PfMAP-2 in vitro and is expressed in a male gametocyte-specific manner in sexual parasite stages^57,58^. Although there is a clear divergence of MAPKs between *Plasmodium* spp. and model organisms, PfMAP-2 as well as its rodent orthologue PbMAP-2 directly or indirectly react to environmental stimuli (e.g. XA, drop in temperature), a feature they share with other eukaryotic members of the ERK1/ERK2 kinase family.

The two MAPKs studied here show peak expression in gametocytes^27,30,33,41,59^, and in the *P. berghei* model parasite it was previously shown that both MAPKs are dispensable for asexual parasite proliferation in mice^37^. In addition, PbMAP-2 was found to be essential for male gametogenesis and further parasite development in the mosquito^27–29^.

Unlike previously suggested^36^, and similar to the situation in *P. berghei*, our results demonstrate that PfMAP-2 is fully dispensable for asexual proliferation of *P. falciparum* parasites. The previous unsuccessful attempt to obtain a PfMAP-2 KO line in 3D7 parasites, a clone of the NF54 strain used here, was based on a single crossover recombination approach that didn’t allow for direct selection of PfMAP-2 KO mutants^36^. We therefore believe that our success in creating PfMAP-2 KO parasites was facilitated by the highly efficient CRISPR/Cas9 system and possibility for direct selection of KO mutants. In addition, *P. falciparum* parasites lacking expression of both MAPKs show no obvious defects in asexual proliferation, sexual commitment and gametocytogenesis, thus revealing the dispensability of both *P. falciparum* MAPKs in these life cycle stages and developmental processes. We can further conclude that the dispensability of PfMAP-1 is not based on upregulation and functional compensation by PfMAP-2 (or vice versa)*.* However, PfMAP-2-depleted parasites show a dramatic reduction in exflagellation similar to what has been observed in *P. berghei* parasites^27–29^. Also, we observed specific expression of PfMAP-2 in late stage male gametocytes, which is in line with previously published proteomics and transcriptomics data^27,41–43^. Moreover, we detected PfMAP-2-GFPDD expression only in late stage gametocytes but not in earlier gametocyte stages and asexual parasites. While Dorin and colleagues also observed PfMAP-2 expression specifically in gametocytes^30^, two other studies detected PfMAP-2 expression in asexual parasites albeit without providing a comparison with gametocyte samples^36,44^. Since we analysed PfMAP-2 expression in a cKD line, where the expression levels of PfMAP-2GFPDD may be lower compared to PfMAP-2 in WT parasites, we might have missed to detect low level PfMAP-2 expression in asexual blood stages, early gametocyte stages and/or female gametocytes. However, our results clearly demonstrate that PfMAP-2 expression is substantially higher and peaks in stage IV and V gametocytes. Our data further show that PfMAP-2 is not essential for gametocytogenesis, especially as in the PfMAP-2-depleted gametocytes protein re-stabilization as late as in mature stage V gametocytes was sufficient to rescue the exflagellation-negative phenotype. Whether PfMAP-2 has important functions in male gametocytes prior to gametogenesis and exflagellation in vivo remains unknown. We also further scrutinized the exflagellation defect and show that upon activation of gametocytes using XA and other stimuli, PfMAP-2-depleted male gametocytes replicate DNA normally, generate octoploid cells and form axonemes. However, upon depletion of PfMAP-2 the replicated nuclear DNA fails to be incorporated into newly forming gametes and microgametocytes do not exflagellate. We therefore conclude that, in accord with the observations on PbMAP-2 function in *P. berghei* parasites, PfMAP-2 is essential for the initiation and/or the process of genome condensation, axoneme beating and cytokinesis. How exactly PfMAP-2 and its putative substrates are involved in these processes needs to be determined in future experiments.

Furthermore, we could show that there is no additive exflagellation defect when both MAPKs are depleted. PfMAP-1_MAP-2 dKO parasites still showed some residual exflagellation comparable to what was observed in PfMAP-2 KO parasites. Hence, during male gametogenesis, PfMAP-1 does not seem to functionally compensate for the loss of PfMAP-2 expression, suggesting clearly distinct functions of the two parasite MAPKs. Hence, the residual exflagellation rates we observed here in *P. falciparum* and others have reported in *P. berghei* PbMAP-2 KO parasites^28,29^ might be explained through partial compensation by a kinase distinct from MAP-1. This putative kinase could either have a substrate specificity overlapping with that of MAP-2 or stochastically phosphorylate substrates essential for the termination of male gametogenesis and exflagellation.

MAPKs phosphorylate a variety of substrates in eukaryotes including the MAPK-activated protein kinases. Amongst other functions, those kinases are involved in gene expression, mRNA stability as well as cell differentiation^49^. Furthermore, in mammals MAPK were shown to directly control gene expression for instance by phosphorylation of transcription factors^34^. In order to identify potential PfMAP-2-dependent regulation of gene expression in male stage V gametocytes as well as gametogenesis, we performed microarray experiments comparing PfMAP-2-stabilizing and -degrading (± Shield-1) as well as gametogenesis activating and non-activating (± XA) conditions. However, we could not identify any transcriptional changes that could be attributed to the function of PfMAP-2 in these stages. We therefore conclude that the role of PfMAP-2 in male gametogenesis is rather based on phosphorylating and changing the activity of target substrates involved in the process of gametogenesis rather than regulating gene expression. In the future, whole cell phosphoproteomics on activated and non-activated stage V gametocytes will hopefully reveal PfMAP-2 substrates and thus provide further details on the molecular processes acting in male gametogenesis.

In summary, we have shown that both *P. falciparum* MAPKs, PfMAP-1 and PfMAP-2, are dispensable for the asexual proliferation of blood stage parasites and for gametocytogenesis. While PfMAP-1 also plays no obvious role in male gametogenesis, PfMAP-2 is specifically expressed in male gametocytes and essential for the crucial process of gametogenesis and exflagellation. Using mosquito feeding assays, the real impact of the exflagellation defect observed in PfMAP-2-depleted parasites in reducing gametocyte transmission could be determined. Such assays would also help clarifying whether the residual exflagellation observed for a small number of PfMAP-2-depleted microgametocytes produces functional microgametes. Furthermore, future work on the transmission potential of PfMAP-1 KO, PfMAP-2 KO and PfMAP-1_MAP-2 dKO parasites might reveal additional functions for the two MAPKs in sexual development and sporogony in the mosquito vector as well as in sporozoites and intra-hepatic schizogony. In *P. berghei*, both MAPKs were shown to be expressed in liver stages suggesting a potential role for MAPK signalling during hepatocyte infection^60^. Obtaining a better understanding of kinase signalling in various parasite stages during the life cycle will broaden our understanding of signalling pathways in these important pathogens and the evolution of kinases in eukaryotes in general. Finally, detailed insight into the function of kinases regulating essential parasite processes may uncover new targets for drug-based malaria interventions that are urgently needed.

## Materials and methods

### Parasite culture

Culturing of *P. falciparum* parasites and synchronization of asexual growth were performed as previously described^61,62^. NF54 asexual parasites were cultured in AB + or B + human RBCs (Blood Donation Center, Zurich, Switzerland) in RPMI-1640 (10.44 g/l), 25 mM HEPES, 100 μM hypoxanthine medium supplemented with 24 mM sodium bicarbonate and 0.5% Albumax II (Gibco). 2 mM choline chloride (CC) was added to the culture medium to prevent unwanted induction of sexual commitment^39^. Cultures were kept at 37 °C and gassed with a mixture of 4% CO_2_, 3% O_2_ and 93% N_2_.

### Transfection constructs

Transgenic parasite lines were generated using the CRISPR/Cas9-based genome editing system. The NF54/MAP-2 KO, NF54/MAP-1 KO and the NF54/MAP-1_MAP-2 dKO cell lines were created using a single plasmid approach. The p_gC plasmid^38^ used for this purpose encodes the Cas9 enzyme and the U6 single gRNA (sgRNA) expression cassette, and the donor sequences for DNA double strand break repair. The PfMAP-1 and PfMAP-2-specific p_gC plasmids (p_gC_*map-1-ko-bsd* and p_gC_*map-2-ko-bsd*) were generated by performing a Gibson assembly reaction using four fragments (1) the *BamHI*- and *EcoRI*-digested p_gC plasmid^38^, (2,3) the *pfmap-1*- or *pfmap-2*-specific 5′ and 3′ homology regions (HRs) amplified from NF54 WT gDNA (primers HR1_M1_F, HR1_M1_R and HR2_M1_F, HR2_M1_R were used to amplify the *pfmap-1-*specific HRs, whereas primers HR1_M2_F, HR1_M2_R and HR2_M2_F, HR2_M2_R were used to amplify the *pfmap-2-*specific HRs) and (4) a resistance cassette (blasticidin S deaminase, BSD), conferring resistance to blasticidin-S-hydrochloride, flanked by the two HRs. The BSD resistance cassette was amplified from pBcam^63^ using primers M1_BSD_F, M1_BSD_R and M2_BSD_F, M2_BSD_R, respectively. These assembled plasmids were subsequently digested with *BsaI* to allow for T4 DNA ligase-dependent insertion of gene-specific sgRNA sequence elements. To generate PfMAP-1 and PfMAP-2 sequence-specific sgRNA elements, complementary oligonucleotides (sgRNA_M1KO_F, sgRNA_M1KO_R and sgRNA_M2KO_F, sgRNA_M2KO_R) were annealed. The resulting double-stranded oligonucleotides carried single-stranded overhangs complementary to the *BsaI*-digested plasmids.

The p_gC_*map-1-ko-hdhfr* plasmid was used to generate the NF54/MAP-1_MAP-2 dKO parasite line by transfecting NF54/MAP-2 KO parasites a second time. This plasmid was generated by assembling three Gibson fragments: (1) The *XbaI-* and *BamHI*-digested p_gC_*map-1-ko-bsd* plasmid retaining the 3′ HR, (2) the *pfmap-1*-specific 5′ HR amplified from NF54 WT gDNA using primers HR1_M1_F and HR1_M1hDHFR_R, and (3) the human dihydrofolate reductase (hDHFR) resistance cassette amplified from pH-gC^38^ using primers hDHFR_F and hDHFR_R.

The NF54/MAP-2GFPDD parasite line was generated using a CRISPR/Cas9-based two-plasmid approach as previously described^38^. The pFdon_*map-2gfpdd* donor plasmid was produced by assembling four fragments in a Gibson reaction using (1) the *HindIII*- and *BamHI*-digested pFdon plasmid^38^, (2,3) the 5′ and 3′ HRs amplified from NF54 WT gDNA using primers HR1_M2KD_F, HR1_M2KD_R and HR2_M2KD_F, HR2_M2KD_R, respectively, and (4) the *gfpdd* sequence amplified from plasmid pHcamGDV1-GFP-DD^38^ using primers GFP_F and GFP_R. The corresponding suicide plasmid pHF_gC_*map-2gfpdd* was generated by annealing the complementary oligonucleotides sgRNA_M2KD_F and sgRNA_M2KD_R and subsequently inserting the resulting double-stranded fragment into the *BsaI-*digested pHF_gC plasmid^38^ by ligation using T4 DNA ligase. Vector maps and gene editing schemes are provided in Fig. 1 and in Supplementary Figs. 1 and 2. Oligonucleotide sequences used for cloning are provided in the Supplementary Table 1.

### Transfection and transgenic cell lines

Transfection of *P. falciparum* parasites with CRISPR/Cas9-based constructs was performed as described previously^38^. NF54 WT parasites were transfected with a total of 100 µg of plasmid DNA (100 µg of p_gC-derived constructs and 50 µg each of pHF_*map-2gfpdd* and pFdon-*map-2gfpdd*). 24 h after transfection, transfected cultures were treated with 2.5 μg/mL blasticidin-S-hydrochloride (for 10 subsequent days) or 4 nM WR99210 (for six subsequent days) according to the resistance cassette transfected (BSD and hDHFR, respectively). After transfection, NF54/MAP-2GFPDD parasites were constantly cultured on 625 nM Shield-1 (+ Shield-1) to stabilize the PfMAP-2GFPDD protein. Stably growing parasite populations were readily obtained 11–21 days post transfection and correct gene editing was confirmed by PCR on gDNA. PCR results are shown in Fig. 1 and in Supplementary Figs. 1 and 2. Primers used to check for correct gene editing are listed in Supplementary Table 2.

### Flow cytometry growth assay

To quantify multiplication of NF54/MAP-1_MAP-2 KO and NF54 WT parasites, flow cytometry measurements of fluorescence intensity were performed. For this purpose, synchronous ring stage (18–24 hpi) parasites were stained using SYBR Green DNA stain (Invitrogen, 1:10,000) for 30 min at 37 °C. Parasites were subsequently washed twice in pre-warmed PBS and measured using the MACS Quant Analyzer 10. Per sample 200,000 cells (unifected RBCs + iRBCs) were analysed. The measurement was repeated at 18–24 hpi in the two subsequent generations. Flow cytometry data were analysed using FlowJo_v10.6.1 and parasitemia was determined by gating of SYBR-positive parasites and determining their proportion among all RBCs.

### Fluorescence microscopy

In order to determine the expression, localisation and depletion of PfMAP-2GFPDD in asexual and sexual parasite stages of the NF54/MAP-2GFPDD parasite line, live cell fluorescence imaging and IFAs were performed as previously described with some minor adaptations^64^. Live cell fluorescence imaging was used to follow PfMAP-2GFPDD expression during gametocytogenesis (stage I–V) and in asexual stages. Nuclei were stained using Hoechst (Merck) at a final concentration of 5 µg/ml and Vectashield (Vector Laboratories) was used as mounting medium.

For IFAs, thin blood smears were fixed in ice-cold methanol or ice-cold methanol/acetone (60:40) for 2 min. To determine the sexual commitment rate of NF54 WT and NF54/MAP-1_MAP-2 dKO parasites, methanol-fixed slides were probed with the primary antibody mouse IgG1 mAb α-Pfs16 (1:100) (kind gift from Robert W. Sauerwein), and the secondary antibody Alexa Fluor 488-conjugated α-mouse (1:250) (Invitrogen #A-11001). To determine the sex-specific expression of PfMAP-2GFPDD and to quantify the male/female ratio, methanol/acetone-fixed cells were probed with primary antibody mAb α-GFP (1:100) (Roche Diagnostics #11814460001), and the rabbit-α-PfG377 serum (1:1,000) (kind gift from Pietro Alano)^47^, and secondary antibodies Alexa Fluor 488-conjugated α-mouse and 564-conjugated α-rabbit IgG (both 1:250) (Invitrogen #A-11001 and #A-11011), respectively. Staining of DNA and mounting of IFA slides was performed using Vectashield with DAPI (Vector Laboratories).

All live cell fluorescence imaging and imaging of methanol and methanol/acetone IFA slides was performed using a Leica DM5000 B fluorescence microscope (20×, 40 × and 63 × objectives) and images were taken using a Leica DFC345 FX camera and the Leica application suite (LAS) software.

IFAs to stain α-tubulin were performed as described^18^. Briefly, RBCs were fixed with 4% paraformaldehyde/0.05% glutaraldehyde in PBS for one hour followed by permeabilisation with 0.1% Triton X-100/PBS for 10 min and blocking with 2% BSA/PBS for two hours. Permeabilised iRBCs were probed with primary mouse anti-α-tubulin (1:1,000) (clone DM1A, Sigma-Aldrich #T9026) and secondary anti-mouse Alexa-488 (1:1,000) (Thermo Fisher Scientific #A28175) antibodies. Nuclei were stained with DAPI (Life Technologies) during incubation with the secondary antibody. Images were acquired with an Axiocam Fluo microscope (Zeiss).

### Western blot

Parasites were obtained by lysis of the RBC membrane using 0.15% saponin/PBS and incubation on ice for 10 min. The obtained parasite pellet was washed 2–3 times in ice-cold PBS. The whole cell protein extract was generated by lysing the parasite pellet in an equal volume of UREA/SDS buffer (8 M Urea, 5% SDS, 50 mM Bis–Tris, 2 mM EDTA, 25 mM HCl, pH 6.5) supplemented with 1 mM DTT and 1 × protease inhibitor cocktail (Merck). For each sample, protein lysate derived from equal parasite numbers were separated on a 3–8% NuPage Tris–Acetate gel using NuPage MES buffer (Novex, Qiagen). The membrane was blocked in 5% milk powder dissolved in 1xPBS/0.1% Tween (PBS-Tween) for 30 min. Proteins were detected using the primary antibodies mouse mAb α-GFP (1:1,000) (Roche Diagnostics #11814460001), or the mouse mAb α-PfGAPDH (1:20,000)^65^, diluted in blocking buffer. The membrane was incubated at 4 °C over night and subsequently washed 3–4 times using PBS-Tween. The secondary antibody α-mouse IgG (H&L)-HRP (GE healthcare #NXA931) was diluted 1:10,000 in blocking buffer and the membrane was incubated for 1–2 h and subsequently washed again 3–4 times using PBS-Tween.

### Induction of sexual commitment and gametocytogenesis

Sexual commitment was induced in synchronized *P. falciparum* parasites (1–1.5% parasitemia) at 18–24 hpi using − SerM medium as described^39^. The − SerM medium was produced by complementing the described RPMI/HEPES culture medium with 24 mM sodium bicarbonate, 0.39% fatty acid-free BSA (Sigma #A6003) and 30 μM each of the two essential fatty acids oleic and palmitic acid (Sigma #O1008 and #P0500, respectively). Parasites cultured on − SerM medium containing 2 mM choline chloride (− SerM/CC), which blocks sexual commitment, were used as a negative control^39^.

To quantify sexual commitment rates, Pfs16 positivity counts were determined for parasites induced for sexual commitment and their matching controls (Fig. 2a). For this purpose, − SerM and − SerM/CC media were removed 30 h after induction (0–6 hpi; generation 2) and replaced with culture medium containing 10% human serum (Blood Donation Centre, Basel, Switzerland) instead of 0.5% Albumax II (+ SerM). Parasites were cultured for another 42 h before thin blood smears were generated at 42–48 hpi and fixed in ice-cold methanol for subsequent α-Pfs16 IFAs. To follow gametocytogenesis, parasites induced for sexual commitment (− SerM medium) were analysed by inspection of Giemsa-stained blood smears, fluorescence live cell imaging and Western blots. − SerM was replaced with + SerM medium 30 h after induction (0–6 hpi, generation 2). Another 24 h later (24–30 hpi; trophozoites and stage I gametocytes), parasites were cultured on + SerM medium supplemented with 50 mM N-acetylglucosamine (+ SerM/GlcNAc) for seven consecutive days to eliminate asexual parasites^66^. Thereafter, gametocytes were cultured using + SerM medium lacking GlcNAc on a heating plate (37 °C) to prevent gametocyte activation.

### Exflagellation assays

Exflagellation assays were performed on day 13 or 14 of gametocytogenesis using mature stage V gametocytes as described^40^. Briefly, gametocyte cultures were pelleted at 400 g for 3 min and the RBC pellet was resuspended in activation medium (+ SerM medium, 100 µM XA) at room temperature/22 °C. After 15 min, the number of exflagellation centres and RBCs per mL of culture were quantified in a Neubauer chamber using bright-field microscopy (40 × objective). Gametocytemia was determined on Giemsa-stained blood smears.

### Flow cytometry analysis of gametocyte DNA content

The DNA content of NF54/MAP-2GFPDD microgametocytes was determined by flow cytometry measurements of fluorescence intensity as described previously^18^. In brief, gametocytes were stained with Vybrant DyeCycle Violet DNA (Thermo Fisher Scientific #V35003) and resuspended in 100 μl of suspended animation medium (RPMI1640 medium containing 25 mM HEPES, 5% fetal calf serum (FCS), 4 mM sodium bicarbonate, pH 7.2). Gamete activation was induced by addition of 100 μl modified exflagellation medium (RPMI 1640 containing 25 mM HEPES, 4 mM sodium bicarbonate, 5% FCS, 200 µM XA, pH 7.8). To rapidly block gametogenesis, 800 μl ice–cold PBS was added at 0 min and 10 min after adding exflagellation medium. Cells were then stained for 30 min at 4 °C with Vybrant DyeCycle Violet DNA (Thermo Fisher Scientific #V35003) and detected using a Beckman Coulter Gallios 4 flow cytometer. Microgametocytes were selected on fluorescence by gating of GFP-positive cells (both ± Shield-1-cultured microgametocytes detectable) (Supplementary Fig. 3). Ploidy was expressed as a percentage of GFP-positive cells and 5,000 cells were analysed per sample.

### Microarray experiments and data analysis

NF54/MAP-2GFPDD stage V gametocytes cultured in presence of Shield-1 were split on day 12 of gametocytogenesis and Shield-1 was removed from one of the paired populations, resulting in samples “stage V ON” and “stage V OFF”. 24 h later (day 13), the paired populations were split again and in one half each gametogenesis was activated by incubation with XA-containing activation medium for 10 min. These samples are termed “stage V act. ON” and “stage V act. OFF”. All cultures (30 mL, 5% haematocrit, approx. 2% gametocytemia) were subsequently spun for 5 min at 2,000 g and the resulting RBC pellet was resuspended in 9 mL Trizol pre-warmed to 37 °C. RNA extraction and cDNA synthesis were performed as described previously^51^. Cy5-labelled sample cDNAs were hybridised against a Cy3-labelled cDNA reference pool prepared from 3D7 WT parasites^67^. Equal amounts of Cy5- and Cy3-labelled cDNA were hybridised for 16 h at 65 °C in an Agilent hybridisation oven (G2545A) on a *P. falciparum* 8 × 15 K Agilent gene expression microarray (GEO platform ID GPL28317), a slightly modified version of the microarray previously published by the Llinas laboratory (GEO platform ID GPL15130)^52^ (one probe removed, 19 probes added) (Supplementary Spreadsheet 1). Slides were scanned using the GenePix scanner 4000B and GenePix pro 6.0 software (Molecular Devices). Microarray data have been deposited in the NCBI Gene Expression Omnibus^68^ and are accessible through GEO Series accession no. GSE147643. The raw microarray data representing relative transcript abundance ratios between each sample and the reference pool (Cy5/Cy3 log2 ratios) were subjected to lowess normalization and background filtering as implemented by the Acuity 4.0 program (Molecular Devices). Flagged features and features with either Cy3 or Cy5 intensities lower than twofold the background were discarded. Log2 ratios for multiple probes per gene were averaged. Transcripts showing expression values in at least seven of the eight samples were included for downstream analysis to identify genes differentially expressed between control (+ Shield-1) and PfMAP-2-depleted (− Shield-1) gametocytes. The heat map shown in Supplementary Fig. 4 was generated using Java Treeview^69^. The Volcano plot shown in Supplementary Fig. 4 was generated using Microsoft Excel. The processed microarray dataset is listed in Supplementary Dataset 1.

## Data availability

The accession number for the microarray data reported in this paper is GEO: GSE147643. Additional data that support the findings of this study are available in Supplementary Dataset 1.

## Supplementary information

Supplementary file1 (PDF 3407 kb)

Supplementary file2 (XLSX 835 kb)

Supplementary file3 (XLSX 15 kb)
