# Supplementary material for: PfMAP-2 is essential for male gametogenesis in the malaria parasite Plasmodium falciparum
Source: Sci Rep. 2020 Jul 17;10:11930. doi: 10.1038/s41598-020-68717-5 (PMC7368081; doi:10.1038/s41598-020-68717-5)
Supplement: Supplementary file 1 — Supplementary file1 (PDF 3407 kb) [file 41598_2020_68717_MOESM1_ESM.pdf]

Supplementary Information

PfMAP-2 is essential for male gametogenesis in the malaria parasite *Plasmodium falciparum*

Eva Hitz[1,2], Aurélia C. Balestra[3], Mathieu Brochet[3], Till S. Voss[1,2,*]

[1]Department of Medical Parasitology and Infection Biology, Swiss Tropical and Public Health Institute, 4051 Basel, Switzerland.

[2]University of Basel, 4001 Basel, Switzerland.

[3]Department of Microbiology and Molecular Medicine, Faculty of Medicine, University of Geneva, 1211 Geneva 4, Switzerland.

[*]Corresponding author: till.voss@swisstph.ch.

This Supplementary Information file includes:

- Supplementary Figures 1-6
- Supplementary Tables 1-2
- Supplementary Dataset 1
- Supplementary Spreadsheet 1

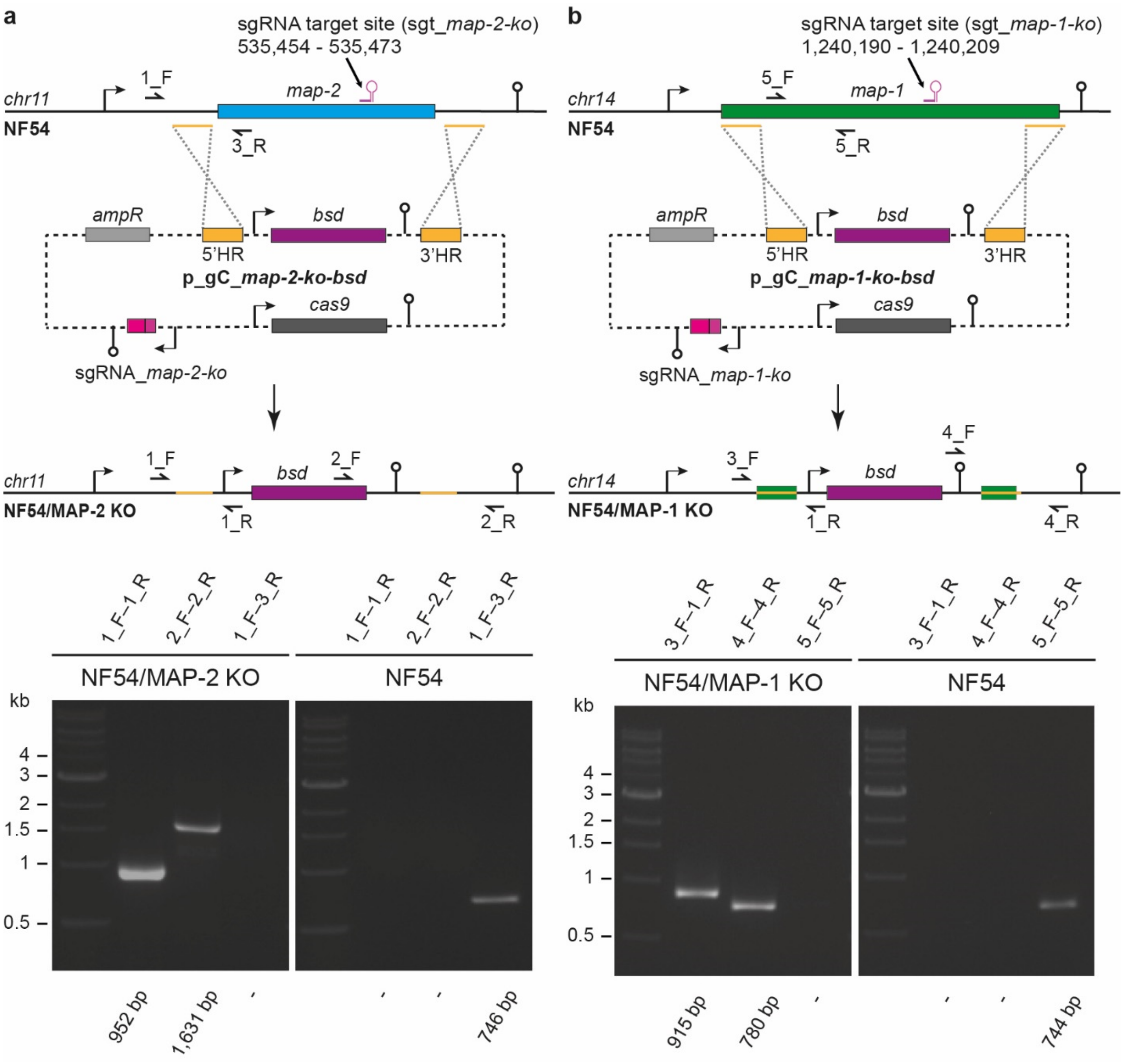

Supplementary Figure 1. Engineering of the PfMAP-1 and PfMAP-2 KO parasite lines. **(a)** Top: Schematic maps of the *pfmap-2* locus, the CRISPR/Cas9 p_gC_*map-2-ko-bsd* plasmid used to generate the NF54/MAP-2 KO parasite line and the disrupted *pfmap-2* locus. Bottom: PCR results on gDNA of NF54/MAP-2 KO parasites show correct editing of the *pfmap-2* locus. PCRs performed on NF54 WT gDNA serve as controls. **(b)** Top: Schematic maps of the *pfmap-1* locus, the CRISPR/Cas9 p_gC_*map-1-ko-bsd* plasmid used to generate the NF54/MAP-1 KO parasite line and the disrupted *pfmap-1* locus. Bottom: PCR results on gDNA of NF54/MAP-1 KO parasites show correct editing of the *pfmap-1* locus. PCRs performed on NF54 WT gDNA serve as controls. NF54, NF54 WT.

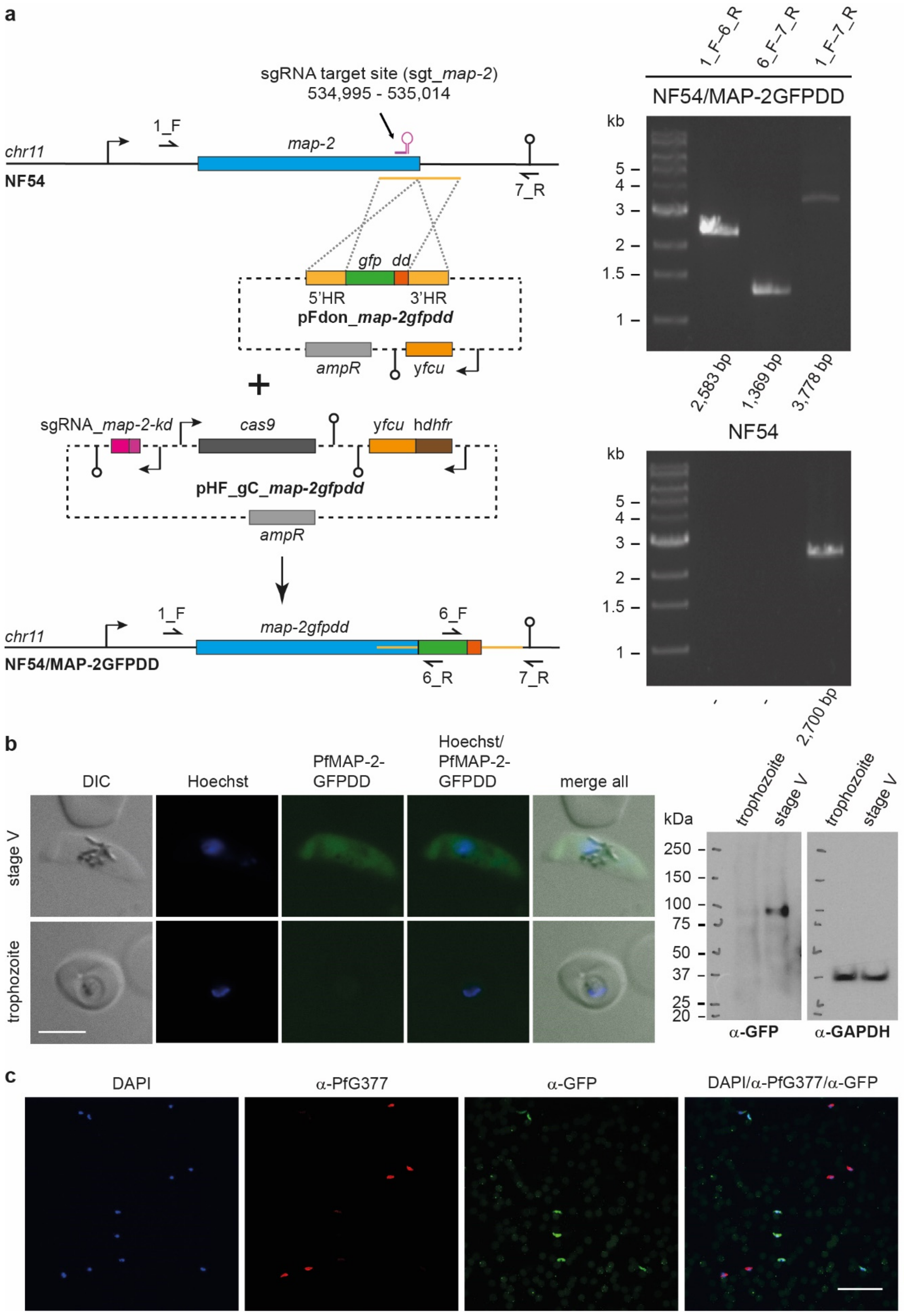

Supplementary Figure 2. Engineering of the NF54/MAP-2GFPDD conditional knockdown parasite line and PfMAP-2 expression. **(a)** Left: Schematic maps of the *pfmap-2* locus, the CRISPR/Cas9 pFdon_*map-2gfpdd* and the pHF_*map-2gfpdd* plasmids used to generate the NF54/MAP-2GFPDD

parasite line and the edited *pfmap-2* locus. Right: PCRs performed on gDNA of NF54/MAP-2GFPDD parasites show correct editing of the *pfmap-2* locus. PCRs performed on NF54 WT (NF54) gDNA serve as controls. **(b)** Expression and localisation of PfMAP-2 in asexual trophozoites (24-30 hpi) and stage V gametocytes assessed by live cell fluorescence imaging and Western blot. Representative live cell fluorescence images are shown. Scale bar = 5 µm. For the Western blot, parasitemia/gametocytemia was determined by inspection of Giemsa-stained blood smears to ensure loading of equal parasite numbers per lane. MW PfMAP-2GFPDD = 97.9 kDa. MW PfGAPDH = 36.6 kDa (control). **(c)** Representative overview images of an α-PfG377/α-GFP IFA experiment on NF54/MAP-2GFPDD stage V gametocytes cultured under protein-stabilizing (+Shield-1) conditions. 200x magnification. Scale bar = 50 µm.

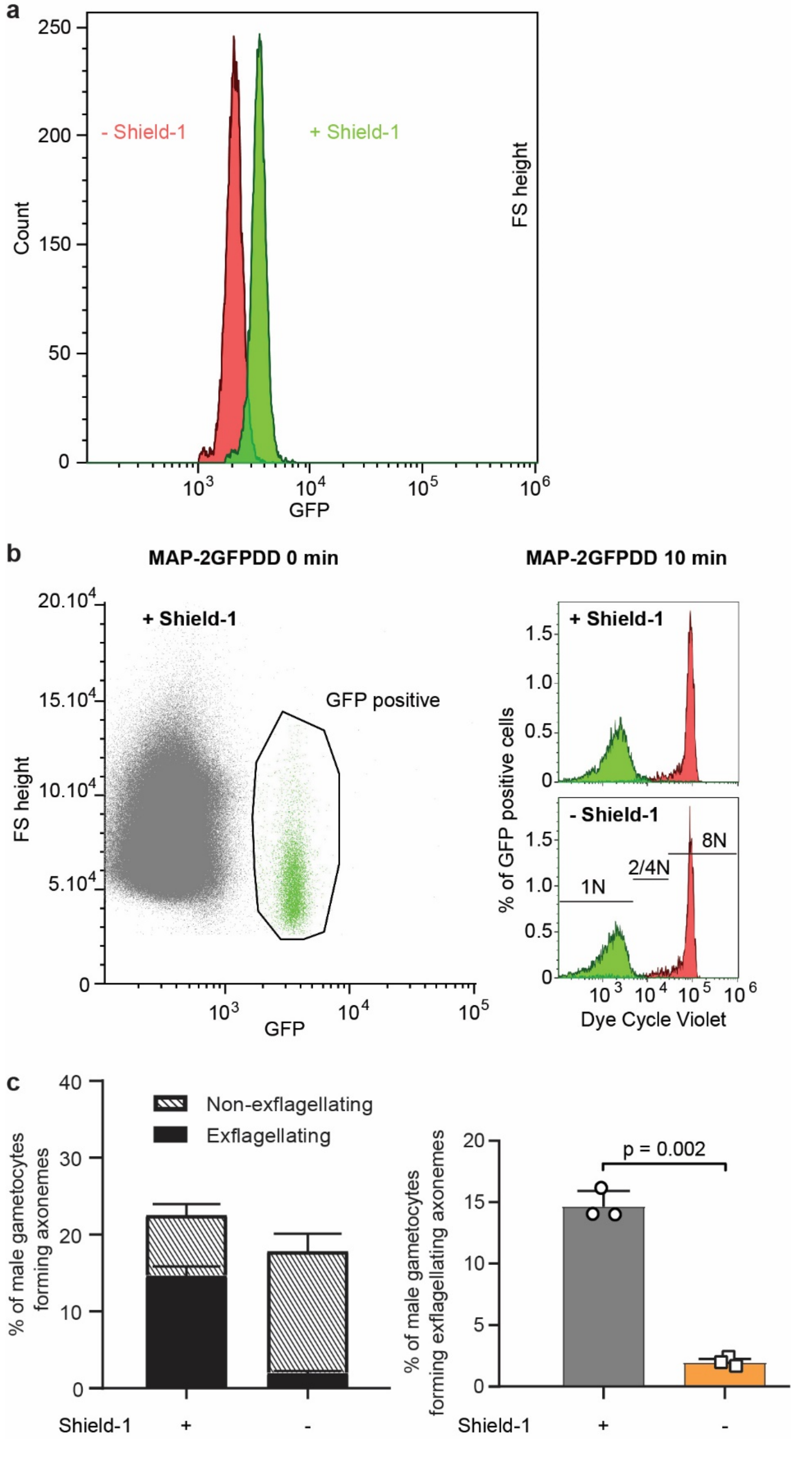

Supplementary Figure 3. Detailed phenotyping of the exflagellation defect in NF54/MAP-2GFPDD parasites. **(a)** Flow cytometry plot showing the differential GFP-fluorescence intensity of NF54/MAP-2GFPDD microgametocytes under protein-degrading (-Shield-1) and protein-stabilizing (+Shield-1) conditions. **(b)** Left: Representative flow cytometry plot showing the fluorescence intensity of individual microgametocytes cultured under protein-stabilizing (+Shield-1) conditions before activation of

gametogenesis (0 min). Vybrant DyeCycle Violet intensity is analysed in the parasite population gated positive for GFP fluorescence. Right: Plots showing the Vybrant DyeCycle Violet intensity and respective ploidy of GFP-positive microgametocytes after (10 min) activation of gametogenesis both under protein-stabilizing (+Shield-1) and protein-degrading (-Shield-1) conditions. **(c)** Left: Plot showing the percentage of microgametocytes forming axonemes and whether or not they exflagellated, determined from anti-α-tubulin IFAs. Right: Percentage of male gametocytes forming exflagellating axonemes after activation of gametogenesis, determined from anti-α-tubulin IFAs. Exflagellating axonemes are defined as axonemes that do not remain around the nucleus. Protein-stabilizing (+Shield-1) and -degrading (-Shield-1) culturing conditions are compared. Parasites were split (±Shield-1) 24 hours before analysis. Values show the mean ±SD of three biological replicates with individual data points represented by open symbols. Significant difference is indicated with the according p-value (paired two-tailed Student's t test).

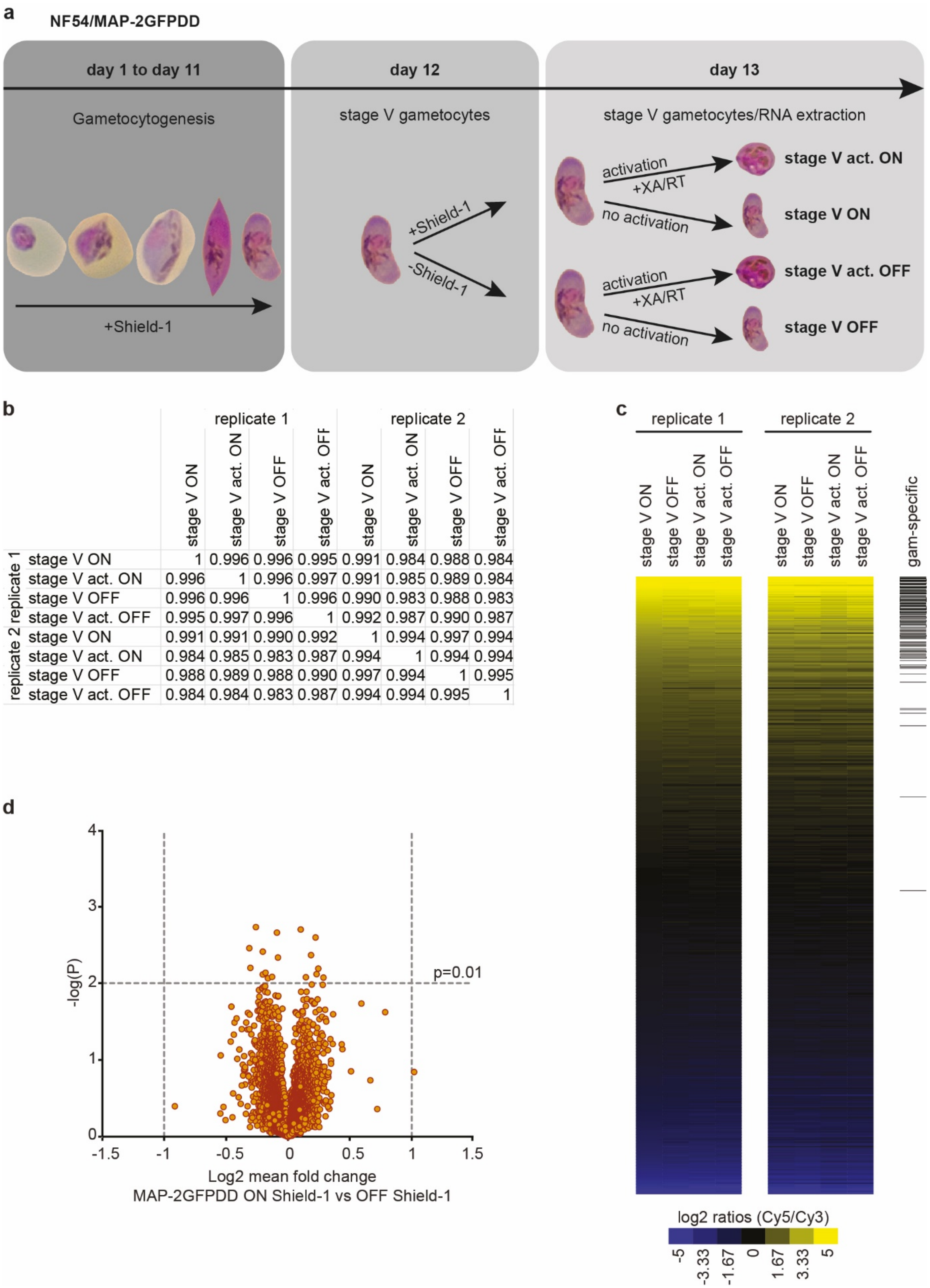

		replicate 1				replicate 2			
		stage V ON	stage V act. ON	stage V OFF	stage V act. OFF	stage V ON	stage V act. ON	stage V OFF	stage V act. OFF
replicate 1	stage V ON	1	0.996	0.996	0.995	0.991	0.984	0.988	0.984
	stage V act. ON	0.996	1	0.996	0.997	0.991	0.985	0.989	0.984
	stage V OFF	0.996	0.996	1	0.996	0.990	0.983	0.988	0.983
	stage V act. OFF	0.995	0.997	0.996	1	0.992	0.987	0.990	0.987
replicate 2	stage V ON	0.991	0.991	0.990	0.992	1	0.994	0.997	0.994
	stage V act. ON	0.984	0.985	0.983	0.987	0.994	1	0.994	0.994
	stage V OFF	0.988	0.989	0.988	0.990	0.997	0.994	1	0.995
	stage V act. OFF	0.984	0.984	0.983	0.987	0.994	0.994	0.995	1

Supplementary Figure 4. Microarray experiments performed using NF54/MAP-2GFPDD stage V gametocytes. **(a)** Schematic of the experimental setup used to generate the non-activated (stage V ON

and stage V OFF) and activated (stage V act. ON and stage V act. OFF) stage V gametocyte samples harvested for microarray experiments. ON, cultured in presence of Shield-1; OFF, cultured in absence of Shield-1; act., XA-induced activation. **(b)** Perason's correlation coefficients for pairwise comparisons of relative mRNA abundances of eight NF54/MAP-2GFPDD microarray samples from two biological replicates. Samples were generated from stage V gametocytes cultured under protein-stabilizing (+Shield-1; ON) and -degrading (-Shield-1; OFF) conditions and before and after XA-induced activation (stage V ON, stage V OFF, stage V act. ON, stage V act. OFF). **(c)** Heat map showing Cy5/Cy3 log2 ratios of all transcripts from the eight NF54/MAP-2GFPDD microarray samples harvested from two biological replicates. Samples were generated from stage V gametocytes cultured under protein-stabilizing (+Shield-1; ON) and -degrading (-Shield-1; OFF) conditions and before and after XA-induced activation (stage V ON, stage V OFF, stage V act. ON, stage V act. OFF). Gametocyte-specific transcripts from a previously published reference dataset[1] are indicated on the right by black horizontal lines. Genes are sorted in descending order according to the Cy5/Cy3 log2 ratio in the stage V ON sample. **(d)** Volcano plot showing the mean fold change in gene expression (log2) between NF54/MAP-2GFPDD gametocytes cultured under protein-stabilizing (+Shield-1; ON) and -degrading (-Shield-1; OFF) conditions and the respective p-values (-log) (unpaired two-tailed Student's t test, equal variance).

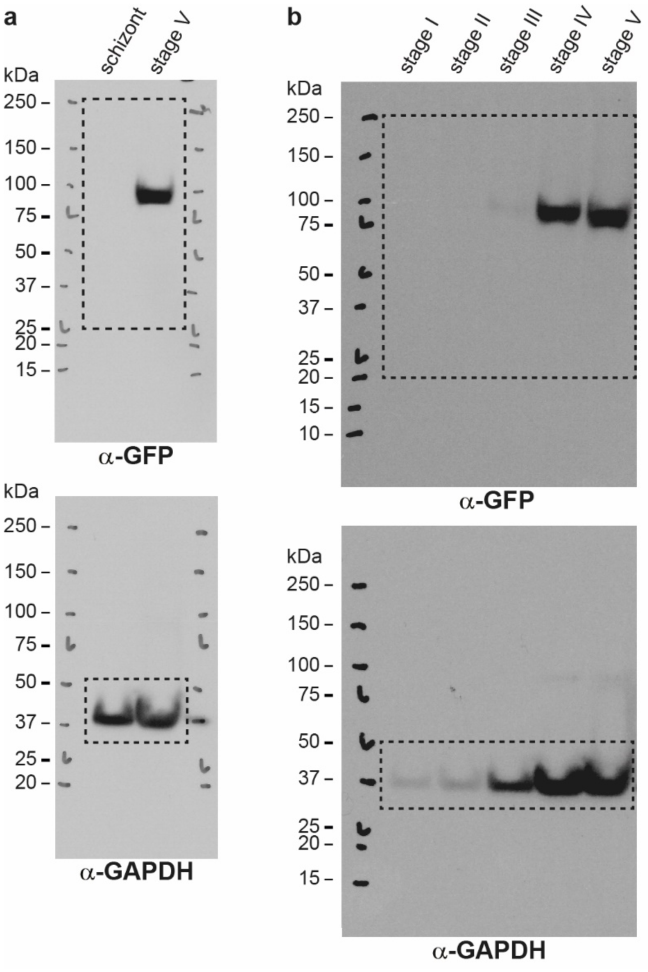

Supplementary Figure 5. Full size Western blots of sections shown in Figure 3. **(a)** Full size Western blots showing the expression of PfMAP-2 in late schizonts (40-48 hpi) and stage V gametocytes. Parasitemia/gametocytemia was determined by inspection of Giemsa-stained blood smears to ensure loading of equal parasite numbers per lane. Dashed lines mark the cropped sections of the blots shown in Figure 3a. MW PfMAP-2GFPDD = 97.9 kDa. MW PfGAPDH = 36.6 kDa (control). **(b)** Full size Western blots showing the expression PfMAP-2 in stage I to stage V gametocytes. Gametocytemia was determined by inspection of Giemsa-stained blood smears to ensure loading of equal parasite numbers per lane. Dashed frames mark the cropped sections of the blots shown in Figure 3b. MW PfMAP-2GFPDD = 97.9 kDa. MW PfGAPDH = 36.6 kDa (control).

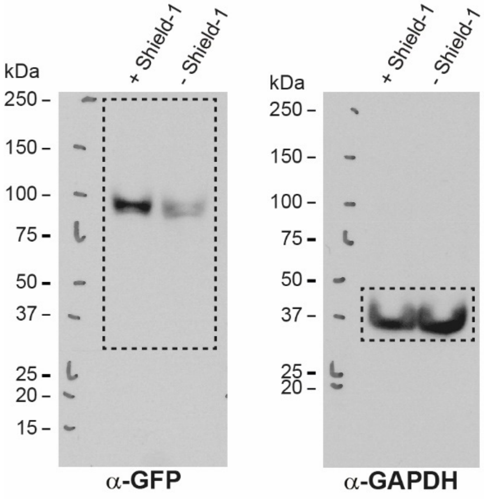

Supplementary Figure 6. Full size Western blot of section shown in Figure 4. Full size Western Blot showing the expression of PfMAP-2 in stage V gametocytes cultured in presence or absence of Shield-1. Parasites were split (±Shield-1) 24 hours prior to the analysis. Dashed frames mark the cropped sections of the blots shown in Figure 4a. MW PfMAP-2GFPDD = 97.9 kDa. MW PfGAPDH = 36.6 kDa (control).

Supplementary Tables

Table 1. Primers used for cloning of CRISPR/Cas9 transfection plasmids. Oligonucleotide names and sequences as well as names of the plasmids and cell lines generated are indicated. Oligonucleotide sequences used to generate PCR fragments for Gibson assembly reactions (Gibson overhangs) and for insertion of gRNA-encoding annealed double-stranded oligonucleotides via T4 DNA ligase-dependent cloning (5' and 3' overhangs) are highlighted with regular and italicized capital letters, respectively.

**Oligonucleotide name**	**Oligonucleotide sequence 5' → 3'**	**Plasmid name**	**Cell line name**
HR1_M1_F	CAGGGTAGCTGATATCGGATCCatgcctaaagaagattgcaagac	p_gC_*map-1-ko-bsd,* p_gC_*map-1-ko-hdhfr*	NF54/MAP-1 KO, NF54/MAP-1_MAP-2 dKO
HR1_M1_R	TTCTATAAATTGAtgctcttaataattgatatatta	p_gC_*map-1-ko-bsd*	NF54/MAP-1 KO, NF54/MAP-1_MAP-2 dKO
HR1_M1hDHFR_R	CCTTTTCTCTTGTgctcttaataattgatatatta	p_gC_*map-1-ko-hdhfr*	NF54/MAP-1 KO, NF54/MAP-1_MAP-2 dKO
HR2_M1_F	TTATTAAATCTAGAggctctttgaaaagcgtaaac	p_gC_*map-1-ko-bsd*	NF54/MAP-1 KO
HR2_M1_R	CAGTGAGCGAGGAAGCGGAAGCTTgtggaaatagtataatgatttg	p_gC_*map-1-ko-bsd,* p_gC_*map-1-ko-hdhfr*	NF54/MAP-1 KO
M1_BSD_F	ATTATTAAGAGCatcaatttatagaaacaaaatatatac	p_gC_*map-1-ko-bsd*	NF54/MAP-1 KO
M1_BSD_R	TTCAAAGAGCCtctagatttaataaatatgttcttata	p_gC_*map-1-ko-bsd*	NF54/MAP-1 KO
hDHFR_F	TTAAGAGCacaagagaaaaggcagaaac	p_gC_*map-1-ko-hdhfr*	NF54/MAP-1_MAP-2 dKO
hDHFR_R	ACGCTTTTCAAAGAGCCTtttaataaatatgttcttatatataatg	p_gC_*map-1-ko-hdhfr*	NF54/MAP-1_MAP-2 dKO
sgRNA_M1KO_F	*AAAC*ctttccttgtatactggttc	p_gC_*map-1-ko-bsd,* p_gC_*map-1-ko-hdhfr*	NF54/MAP-1 KO, NF54/MAP-1_MAP-2 dKO
sgRNA_M1KO R	*TATT*gaaccagtatacaaggaaag	p_gC_*map-1-ko-bsd,* p_gC_*map-1-ko-hdhfr*	NF54/MAP-1 KO, NF54/MAP-1_MAP-2 dKO
HR1_M2_F	CAGGGTAGCTGATATCGcatattgaaccttcctatttc	p_gC_*map-2-ko-bsd*	NF54/MAP-2 KO
HR1_M2_R	GTTTCTATAAATTGATgattttactgagaggttaac	p_gC_*map-2-ko-bsd*	NF54/MAP-2 KO
HR2_M2_F	TTATTAAATCTAGAgaaggaatgaaggaatatttc	p_gC_*map-2-ko-bsd*	NF54/MAP-2 KO
HR2_M2_R	CAGTGAGCGAGGAAGCGGAatttattcaagcgggacac	p_gC_*map-2-ko-bsd*	NF54/MAP-2 KO
M2_BSD_F	CTCAGTAAAATCatcaatttatagaaacaaaatatatac	p_gC_*map-2-ko-bsd*	NF54/MAP-2 KO
M2_BSD_R	CCTTCATTCCTTCtctagatttaataaatatgttcttata	p_gC_*map-2-ko-bsd*	NF54/MAP-2 KO
sgRNA_M2KO_F	*AAAC*ttatttcctggatcttcttg	p_gC_*map-2-ko-bsd*	NF54/MAP-2 KO
sgRNA_M2KO_R	*TATT*caagaagatccaggaaataa	p_gC_*map-2-ko-bsd*	NF54/MAP-2 KO
HR1_M2KD_F	CGAGTCAGTGAGCGAGGAttatcacctgatcataattc	pFdon_*map-2-gfpdd*	NF54/MAP-2GFPDD
HR1_M2KD_R	CTTTTCTGGTGGATGTTGAGtttcgctggtataattaaatcagcatgaaatg	pFdon_*map-2-gfpdd*	NF54/MAP-2GFPDD
HR2_M2KD_F	ACTGGAATGAgcataatttttatctatac	pFdon_*map-2-gfpdd*	NF54/MAP-2GFPDD
HR2_M2KD_R	AGGCCCTTTTCTCTTGTGtaatttattcaagcgggacac	pFdon_*map-2-gfpdd*	NF54/MAP-2GFPDD
GFP_F	CTCAACATCCACCAGAAAAGtttctacaatatgggatccagtggaatgagtaaag	pFdon_*map-2-gfpdd*	NF54/MAP-2GFPDD
GFP_R	GATAAAAATTATGCtcattccagttttagaagctc	pFdon_*map-2-gfpdd*	NF54/MAP-2GFPDD
sgRNA_M2KD_F	*AAAC*gcaaagttaaatatacatca	pHF_*map-2-gfpdd*	NF54/MAP-2GFPDD
sgRNA_M2KD_R	*TATT*tgatgtatatttaactttgc	pHF_*map-2-gfpdd*	NF54/MAP-2GFPDD

Table 2. Primers used for PCRs on gDNA of transgenic parasite lines. Oligonucleotide names and sequences as well as the names of the cell lines from which gDNA was extracted to perform integration PCRs are indicated.

Primer name	Primer sequence 5' → 3'	Cell line name
1_F	atatctactgtctccatatac	NF54/ MAP-2 KO; NF54/MAP-1_MAP-2 dKO; NF54/MAP-2GFPDD
1_R	cattgtttaatactactacatg	NF54/ MAP-2 KO; NF54/ MAP-1 KO; NF54/MAP-1_MAP-2 dKO
2_F	gtatattttaaactagaaaagg	NF54/ MAP-2 KO; NF54/MAP-1_MAP-2 dKO
2_R	tgactaagacatgcataagag	NF54/ MAP-2 KO; NF54/MAP-1_MAP-2 dKO
3_F	ccacaacaacatttatcatt	NF54/ MAP-2 KO; NF54/MAP-1_MAP-2 dKO
3_R	tcatccctactactagactc	NF54/ MAP-1 KO; NF54/MAP-1_MAP-2 dKO
4_F	catgttttgtaatttatgggatagcg	NF54/ MAP-1 KO; NF54/MAP-1_MAP-2 dKO
4_R	gatagttttacaacggttcag	NF54/ MAP-1 KO; NF54/MAP-1_MAP-2 dKO
5_F	gttgggtagtacgcattatac	NF54/ MAP-1 KO; NF54/MAP-1_MAP-2 dKO
5_R	agaactacatgctctaggttg	NF54/ MAP-1 KO; NF54/MAP-1_MAP-2 dKO
6_F	ggttatgtacaggaaagaac	NF54/MAP-2GFPDD
6_R	gtgtgagttatagttgtattcc	NF54/MAP-2GFPDD
7_R	tcatccctactactagactc	NF54/MAP-2GFPDD

Dataset 1. Processed microarray data obtained from NF54/MAP-2GFPDD stage V gametocytes. Columns A-B: Gene ID and gene annotation (www.plasmodb.org; v41). Column C: gametocyte-specific genes[1]. Columns D-K: Cy5/Cy3 log2 ratios for all transcripts in the eight samples harvested from two biological replicates from NF54/MAP-2GFPDD stage V gametocytes cultured in presence (columns D-G) or absence (columns H-K) of Shield-1 before (columns D, E, H, I) and after (columns F, G, J, K) XA-induced activation. Column L: mean fold change in gene expression (log2) between NF54/MAP-2GFPDD gametocytes cultured in presence and absence of Shield-1. Column M: p-value (unpaired two-tailed Student's t test, equal variance). Columns N-Q: fold change in gene expression (log2) between NF54/MAP-2GFPDD gametocytes cultured in presence and absence of Shield-1 for each replicate experiment performed before and after XA-induced activation. ON, cultured in presence of Shield-1; OFF, cultured in absence of Shield-1; act., XA-induced activation.

Spreadsheet 1. Comparison of the GEO microarray platforms GPL15130 and GPL28317. Worksheet 1: Comparison of the overall design and probe content of the *P. falciparum* Agilent microarray platforms GPL15130 (Agilent-037237)[2] and GPL28317 (Agilent-085039) (this study). Worksheet 2: Names (column 1), IDs (column 2) and sequences (column 3) of the oligonucleotide probes that differ between the *P. falciparum* Agilent microarray platforms GPL15130 (Agilent-037237) [2] and GPL28317 (Agilent-

085039) (this study). *P. falciparum* codon-optimised marker gene sequences for which probes have been included in the GPL28317 (Agilent-085039) microarray (column D).

References

1 Young, J. A. *et al.* The Plasmodium falciparum sexual development transcriptome: a microarray analysis using ontology-based pattern identification. *Mol Biochem Parasitol* **143**, 67-79, doi:10.1016/j.molbiopara.2005.05.007 (2005).

2 Painter, H. J., Altenhofen, L. M., Kafsack, B. F. & Llinas, M. Whole-genome analysis of Plasmodium spp. Utilizing a new agilent technologies DNA microarray platform. *Methods Mol. Biol* **923**, 213-219, doi:10.1007/978-1-62703-026-7_14 (2013).
